# Associations between macrolide antibiotics prescribing during pregnancy and adverse child outcomes in the UK: population based cohort study

**DOI:** 10.1136/bmj.m331

**Published:** 2020-02-19

**Authors:** Heng Fan, Ruth Gilbert, Finbar O’Callaghan, Leah Li

**Affiliations:** 1 Population, Policy and Practice Programme, Great Ormond Street Institute of Child Health, University College London, London WC1N 1EH, UK; 2 Developmental Neurosciences Programme, Great Ormond Street Institute of Child Health, University College London, London, UK

## Abstract

**Objective:**

To assess the association between macrolide antibiotics prescribing during pregnancy and major malformations, cerebral palsy, epilepsy, attention deficit hyperactivity disorder, and autism spectrum disorder in children.

**Design:**

Population based cohort study.

**Setting:**

The UK Clinical Practice Research Datalink.

**Participants:**

The study cohort included 104 605 children born from 1990 to 2016 whose mothers were prescribed one macrolide monotherapy (erythromycin, clarithromycin, or azithromycin) or one penicillin monotherapy from the fourth gestational week to delivery. Two negative control cohorts consisted of 82 314 children whose mothers were prescribed macrolides or penicillins before conception, and 53 735 children who were siblings of the children in the study cohort.

**Main outcome measures:**

Risks of any major malformations and system specific major malformations (nervous, cardiovascular, gastrointestinal, genital, and urinary) after macrolide or penicillin prescribing during the first trimester (four to 13 gestational weeks), second to third trimester (14 gestational weeks to birth), or any trimester of pregnancy. Additionally, risks of cerebral palsy, epilepsy, attention deficit hyperactivity disorder, and autism spectrum disorder.

**Results:**

Major malformations were recorded in 186 of 8632 children (21.55 per 1000) whose mothers were prescribed macrolides and 1666 of 95 973 children (17.36 per 1000) whose mothers were prescribed penicillins during pregnancy. Macrolide prescribing during the first trimester was associated with an increased risk of any major malformation compared with penicillin (27.65 *v* 17.65 per 1000, adjusted risk ratio 1.55, 95% confidence interval 1.19 to 2.03) and specifically cardiovascular malformations (10.60 *v* 6.61 per 1000, 1.62, 1.05 to 2.51). Macrolide prescribing in any trimester was associated with an increased risk of genital malformations (4.75 *v* 3.07 per 1000, 1.58, 1.14 to 2.19, mainly hypospadias). Erythromycin in the first trimester was associated with an increased risk of any major malformation (27.39 *v* 17.65 per 1000, 1.50, 1.13 to 1.99). No statistically significant associations were found for other system specific malformations or for neurodevelopmental disorders. Findings were robust to sensitivity analyses.

**Conclusions:**

Prescribing macrolide antibiotics during the first trimester of pregnancy was associated with an increased risk of any major malformation and specifically cardiovascular malformations compared with penicillin antibiotics. Macrolide prescribing in any trimester was associated with an increased risk of genital malformations. These findings show that macrolides should be used with caution during pregnancy and if feasible alternative antibiotics should be prescribed until further research is available.

**Trial registration:**

ClinicalTrials.gov NCT03948620

## Introduction

Macrolide antibiotics (including erythromycin, clarithromycin, and azithromycin) are among the most frequently prescribed antibiotics during pregnancy in Western countries.^1^ ^2^ ^3^ Policy advice about macrolide use in pregnancy varies. A warning against the use of erythromycin during the first trimester was issued in Sweden in 2005 after a reported association between macrolides and cardiac malformations.^4^ ^5^ The United Kingdom Medicines and Healthcare products Regulatory Agency advise that alternatives to clarithromycin and azithromycin should be prescribed during pregnancy.^6^ Warnings have been issued in the United States and the UK against the use of azithromycin and clarithromycin in adults with a high risk of cardiovascular complications,^7^ ^8^ based on evidence of an increase in the risk of cardiac arrhythmias and cardiac mortality (two systematic reviews, 19 randomised controlled trials).^9^ ^10^ A recent systematic review on the use of macrolides during pregnancy showed consistent evidence of an increased risk of miscarriage, but less consistent evidence for congenital malformations, cerebral palsy, and epilepsy.^11^

We conducted a large, retrospective cohort study with data from a UK representative primary care database to address these uncertainties. We compared children born to mothers prescribed macrolide antibiotics during pregnancy with those whose mothers were prescribed penicillins to minimise the effects of confounding because of infection. Macrolides are often used as alternatives for patients with penicillin allergy; penicillins have long established safety records during pregnancy.^2^ ^6^ ^12^ We hypothesised that macrolides might induce fetal cardiac arrhythmia and short term fetal hypoxia,^13^ ^14^ ^15^ and so we compared major malformations and neurodevelopmental disorders in children that could result from short term fetal hypoxia.

## Methods

### Study population

We used records from the Clinical Practice Research Datalink (CPRD), a large anonymised primary care database that covers 6.9% of the UK population.^16^ CPRD is broadly representative of the national population in terms of age, sex, and ethnicity. The database comprises records of consultations, diagnoses and symptoms, tests, referrals to and feedback from secondary care, health related behaviours, and additional care administered as part of routine general practice. Prescription data are automatically recorded when prescriptions are issued.^16^ In the UK, general practices are the main point of contact for non-emergency medical care, including pregnancy, and CPRD has been extensively used in pharmacoepidemiology studies in pregnancy.^17^ ^18^ ^19^ ^20^ We report our findings according to the REporting of studies Conducted using Observational Routinely collected Data (RECORD) guideline (supplementary table S1).^21^

We used the mother-baby link on CPRD to create a cohort of all babies who were born alive in the UK from January 1990 to June 2016,^22^ and estimated the date of the last menstrual period for all mothers (supplementary text S1). We included children registered with the general practice within 6 months of birth whose mothers were aged 14-50 years and registered with a CPRD practice from at least 50 weeks before the estimated last menstrual period. We excluded children with known chromosomal abnormalities and children whose mothers were prescribed a known teratogenic drug during pregnancy (warfarin, angiotensin converting enzyme inhibitors, antineoplastic agents, isotretinoin, misoprostol, or thalidomide). Children were followed from birth to 14 years, death, or end of follow-up (June 2016), whichever came first.

The Independent Scientific Advisory Committee for Medicines and Healthcare products Regulatory Agency Database Research approved the study protocol (19_038R2). We registered the study on ClinicalTrials.gov (NCT03948620).

### Prescriptions

**Fig 1:**
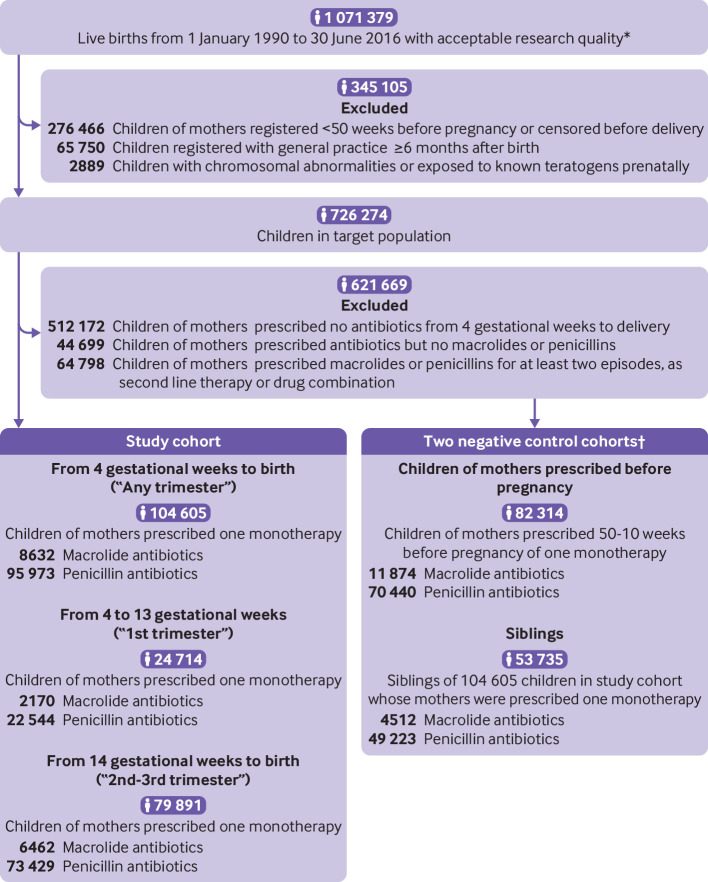
Flowchart of study cohort. Mothers could have had more than one pregnancy. *Acceptable patients were those who met the Clinical Practice Research Datalink (CPRD) threshold for data quality in general practices defined as contributing up to standard data. †9484 children were included in both negative control cohorts

We included children whose mothers were prescribed one episode of macrolide monotherapy or one episode of penicillin monotherapy between four gestational weeks and delivery (hereafter, any trimester). One episode of monotherapy refers to one or more consecutive prescriptions for a single antibiotic separated by no more than 30 days and uninterrupted by prescriptions for other antibiotic drugs. We started the time window at four gestational weeks to evaluate all prescriptions from five gestational weeks, the start of organogenesis, and allowed for a typical prescription of one week. We further divided the time window into four gestational weeks to 13 gestational weeks (first trimester), the critical period for most major malformations, and 14 gestational weeks to birth (second to third trimester).^23^ We created two negative control cohorts: firstly, children of mothers prescribed one macrolide or penicillin monotherapy 50-10 weeks before the last menstrual period and not included in the study cohort; and secondly, siblings of the children in the study cohort. Prescriptions of macrolides and penicillins were identified using a drug code list based on the British National Formulary (chapters 5.1.5 and 5.1.1). The index date of exposure is the date of the first prescription of the monotherapy (fig 1).

### Outcomes

The main outcomes were major malformations (any and system specific) and four neurodevelopmental disorders. Major malformations were identified from 11 system specific malformations defined by the European Surveillance of Congenital Anomalies (EUROCAT).^24^ We included five system specific malformations (nervous, cardiovascular, gastrointestinal, genital, and urinary) which met prespecified power criteria using the EUROCAT prevalence table.^25^ Supplementary table S2 and text S2 report further details. We present the most frequent codes recorded for each system specific malformation in supplementary table S3.

Neurodevelopmental disorders (cerebral palsy, epilepsy, attention deficit hyperactivity disorder, and autism spectrum disorder) were defined as the time to the first diagnostic or treatment code that indicated the outcome by 14 years old. We used the random forest approach to identify children who potentially had cerebral palsy based on informative prescription or Read codes, as previously described.^26^ A paediatric neurologist (FOC) who was blinded to prenatal antibiotic exposure validated these potential cases. We identified other neurodevelopmental disorders (epilepsy, attention deficit hyperactivity disorder, and autism spectrum disorder) by using previously validated criteria and diagnostic codes or prescriptions (supplementary table S2).

### Covariates

Macrolides are widely used as alternatives for women with suspected allergy to penicillins,^6^ therefore comparison with penicillins could minimise confounding because of infection. However, residual confounding might exist if macrolides were prescribed for specific indications (eg, chlamydia), or when potential risk factors for malformations or neurodevelopmental outcomes differed between treatment groups.

We included the following covariates: maternal characteristics at conception and chronic risk factors (age at delivery, calendar year of delivery, alcohol misuse, illicit drug use, tobacco use, obesity, hypertension, diabetes, anxiety, depression, and epilepsy); and pregnancy related variables (parity, multiple birth, chronic medical treatments, genitourinary tract infections and sexually transmitted infections during pregnancy). Genitourinary tract infections and sexually transmitted infections are potentially associated with preterm labour (or congenital malformations) and we adjusted for these events as confounders (defined in supplementary table S4).^27^

### Statistical analysis

We derived the standardised difference (>0.1 as meaningful imbalances) to assess covariate balance between the macrolide and penicillin groups.^28^ For malformations, we calculated absolute risks (per 1000 children) and risk ratios with 95% confidence intervals using log binomial models. For neurodevelopmental disorders for which the follow-up time could be censored, we estimated absolute rates (per 1000 person years) and hazard ratios with 95% confidence intervals by using Cox proportional hazard models. We tested the proportional hazard assumption by using Schoenfeld residuals after all Cox proportional hazard models had been calculated. Robust standard errors were estimated to account for the clustering of siblings and multiple births in mothers.

We adjusted models for covariates by using the following method. We derived the propensity score (predicted probability of macrolides *v* penicillins) with logistic regression, divided the propensity score of the macrolide group into 50 strata, and weighted children in the penicillin group in each stratum according to the distribution of the propensity score of the macrolide group.^29^

The primary analysis compared children of mothers prescribed one macrolide monotherapy or one penicillin monotherapy during the first trimester, the second to third trimester, and any trimester. We performed subgroup analyses by macrolide subtype because studies in non-pregnant adults suggest that clarithromycin and azithromycin could have a stronger arrhythmic effect than erythromycin,^7^ ^8^ and azithromycin is more likely to be indicated for sexually transmitted infections. We also analysed treatment duration (less than seven days and seven days or longer, macrolides *v* penicillins) to evaluate a dose dependent effect of macrolides.

### Sensitivity analysis

We evaluated potential confounding by unmeasured familial characteristics. Firstly, we conducted a negative control analysis in mothers prescribed either macrolides or penicillins 50-10 weeks before their last menstrual period. Secondly, we performed a negative control analysis among siblings. We compared the risks of adverse outcomes between siblings of children in the study cohort whose mothers were prescribed macrolides and siblings of children in the study cohort whose mothers were prescribed penicillins. A null finding in these two analyses would provide indirect evidence of no substantial residual confounding owing to differences in family related factors between mothers prescribed macrolides and those prescribed penicillins (eg, maternal socioeconomic status and genetic factors).

Thirdly, to mitigate confounding by infection, we restricted analyses to children whose mothers were prescribed antibiotics for respiratory tract infections (prescribed within six days of diagnosis); we expected comparable or larger effect sizes than those obtained in the primary analysis. Respiratory tract infections are one of the most common indications for antibiotic prescriptions. When these infections are diagnosed in primary care, they are usually self-limiting with viral causes. Therefore, any benefit (in terms of reduced risk of adverse outcomes in children) from antibiotic treatment of bacterial respiratory tract infections would be small or negligible, and the chance of detecting potential adverse effects of antibiotic treatment would be increased. Fourthly, we performed probabilistic multiple bias analyses to quantify the potential bias caused by outcome misclassification and live birth bias. Live birth bias might occur because we did not include pregnancies in the cohort that resulted in fetal deaths, which could occur disproportionately in women prescribed macrolides and in those prescribed penicillins.****

As a post hoc analysis, we assessed whether common specific malformations were associated with maternal prescribing of macrolides versus penicillins. We only analysed outcomes when at least five children were affected in the macrolides group. All analyses were conducted in RStudio version 3.5.1.

### Patient and public involvement

This study was based on secondary analyses of an administrative database and did not directly involve patient participants. We did not involve patients in designing the research question, outcome measures, or interpretation or writing up of results of this study.

## Results

**Fig 2:**
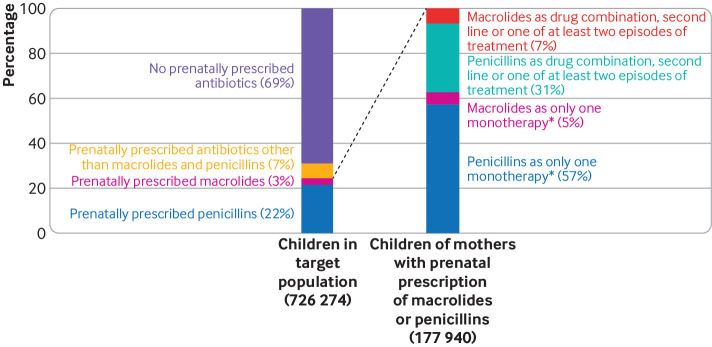
Antibiotic prescriptions during pregnancy in this study. *9698 and 101 969 children were prenatally exposed to only one monotherapy of macrolides and penicillins, respectively. Study cohort included 8632 and 95 973 children exposed to antibiotics between four gestational weeks and birth (fig 1)

Mothers of 31% of children in the target population were prescribed at least one antibiotic during pregnancy. Penicillins and macrolides accounted for about 69% and 10% of the prescriptions, respectively, with an average of 62% as the single monotherapy during pregnancy (fig 1 and fig 2).****

The study cohort included 104 605 children, of whom 8632 (8.3%) were born to mothers prescribed one macrolide monotherapy and 95 973 (91.7%) were born to mothers prescribed one penicillin monotherapy during pregnancy. The children were followed for a median of 5.8 years (interquartile range 2.4-12.1 years) after birth. In one negative control cohort, 82 314 children were born to mothers prescribed macrolides (n=11 874) or penicillins (n=70 440) before conception. In the second negative control cohort, 53 735 children were identified as siblings of children in the study cohort who were prenatally exposed to macrolides (n=4512) or penicillins (n=49 223; fig 1).

Maternal characteristics were similar in the macrolide antibiotic group and the penicillin antibiotic group. Characteristics were also similar according to time of prescription: during the first trimester, second to third trimester, or 50-10 weeks before pregnancy, with differences balanced after propensity score adjustment (table 1 and supplementary tables S5-S7).

**Table 1:** Unadjusted and propensity score adjusted baseline characteristics of children whose mothers were prescribed macrolides or penicillins from four to 13 gestational weeks (first trimester). Values are numbers (percentages) unless indicated otherwise

Characteristics	Unadjusted				Propensity score adjusted*		
	Macrolides	Penicillins	St.diff		Macrolides	Penicillins	St.diff
No of children	2170	22 544	—		2170	22 509.8	—
Maternal baseline characteristics							
Age at delivery:	—	—	0.108		—	—	0.008
13-19	130 (6.0)	986 (4.4)	—		130 (6.0)	1372.2 (6.1)	—
20-24	377 (17.4)	3510 (15.6)	—		377 (17.4)	3881.9 (17.2)	—
25-29	524 (24.1)	6223 (27.6)	—		524 (24.1)	5437.1 (24.2)	—
30-34	664 (30.6)	6892 (30.6)	—		664 (30.6)	6941.3 (30.8)	—
35-50	475 (21.9)	4933 (21.9)	—		475 (21.9)	4877.3 (21.7)	—
Calendar year of delivery:	—	—	0.115		—	—	0.016
1990-94	170 (7.8)	2225 (9.9)	—		170 (7.8)	1843.0 (8.2)	—
1995-99	318 (14.7)	3746 (16.6)	—		318 (14.7)	3323.8 (14.8)	—
2000-04	496 (22.9)	4404 (19.5)	—		496 (22.9)	5175.9 (23.0)	—
2005-09	568 (26.2)	5594 (24.8)	—		568 (26.2)	5781.4 (25.7)	—
2010-16	618 (28.5)	6575 (29.2)	—		618 (28.5)	6385.8 (28.4)	—
Alcohol misuse	129 (5.9)	1047 (4.6)	0.058		129 (5.9)	1353.6 (6.0)	0.003
Illicit drug use	31 (1.4)	243 (1.1)	0.032		31 (1.4)	323.6 (1.4)	0.001
Tobacco use	790 (36.4)	7412 (32.9)	0.074		790 (36.4)	8168.1 (36.3)	0.002
Obesity	262 (12.1)	2578 (11.4)	0.020		262 (12.1)	2742.7 (12.2)	0.003
Hypertension	161 (7.4)	1623 (7.2)	0.008		161 (7.4)	1655.9 (7.4)	0.002
Diabetes	68 (3.1)	782 (3.5)	0.019		68 (3.1)	710.3 (3.2)	0.001
Anxiety	74 (3.4)	556 (2.5)	0.056		74 (3.4)	763.1 (3.4)	0.001
Depression	227 (10.5)	2288 (10.1)	0.01		227 (10.5)	2381.0 (10.6)	0.004
Epilepsy	25 (1.2)	155 (0.7)	0.049		25 (1.2)	238.4 (1.1)	0.009
Pregnancy related characteristics							
Parity ≥1	782 (36.0)	8080 (35.8)	0.004		782 (36.0)	8070.8 (35.9)	0.004
Multiple births	52 (2.4)	535 (2.4)	0.002		52 (2.4)	564.5 (2.5)	0.007
Genitourinary tract infection	90 (4.1)	2796 (12.4)	0.303		90 (4.1)	889.2 (4.0)	0.010
Sexually transmitted infection	102 (4.7)	301 (1.3)	0.198		102 (4.7)	978.2 (4.3)	0.017
Treatment of chronic medical conditions	422 (19.4)	4066 (18.0)	0.036		422 (19.4)	4388.4 (19.5)	0.001

* Exposure propensity scores were measured as predicted probability of receiving macrolides versus penicillins, conditional on maternal and pregnancy related characteristics included in this table. Fifty strata were created based on distribution of propensity score of macrolides group. Weights for penicillin group were calculated according to distribution of macrolide group among strata and were used to estimate adjusted baseline characteristics. A meaningful between group imbalance was assessed by absolute standardised difference (St.diff=difference in means in units of standard deviation) of more than 0.1. Numbers in adjusted penicillin group were non-integer because they were weighted based on distribution of propensity score of macrolide group.

### Primary analysis

**Fig 3:**
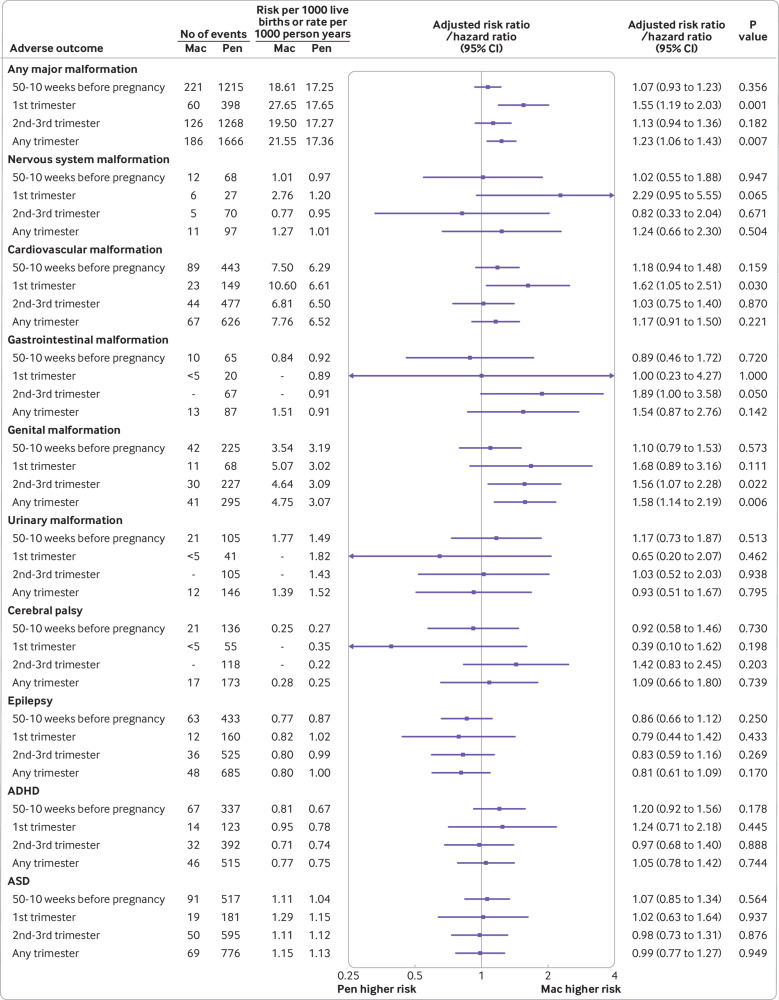
Association between adverse child outcomes and macrolide (*v* penicillin) antibiotics prescribing before or during pregnancy by timing of prescription: 50-10 weeks before pregnancy; during first trimester (from****four to****13 gestational weeks); during second to third trimester (from 14 gestational weeks to delivery); and during any trimester (from four gestational weeks to delivery). To protect confidentiality of patient data, <5 is given for less than five events, and - is given to avoid deduction. ADHD=attention deficit hyperactivity disorder; ASD=autism spectrum disorder; Mac=macrolide; Pen=penicillin

The prevalence of major malformations was 27.7 per 1000 livebirths in mothers prescribed macrolides in the first trimester, and 19.5 per 1000 livebirths in mothers prescribed macrolides in the second to third trimester. Equivalent rates in the penicillin group were stable (17.7 and 17.3 per 1000 livebirths, respectively). Macrolide prescribing in the first trimester was associated with increased risk of any malformation (adjusted risk ratio 1.55, 95% confidence interval 1.19 to 2.03), and specifically, cardiovascular malformations (10.6 *v* 6.6 per 1000 livebirths; adjusted risk ratio 1.62, 95% confidence interval 1.05 to 2.51; fig 3).

Macrolide prescribing during the second to third trimester showed no increased risk of any major malformation (adjusted risk ratio 1.13, 95% confidence interval 0.94 to 1.36). However, we observed a borderline association with gastrointestinal malformations (1.89, 1.00 to 3.58). Macrolide prescribing in any trimester was associated with an increased risk of genital malformations (1.58, 1.14 to 2.19; mainly hypospadias, as we show in the post hoc analyses in supplementary table S12). We found no association between the four neurodevelopmental disorders and macrolide prescribing during pregnancy (fig 3).

Analyses of subtypes of macrolide were limited owing to few events. Erythromycin prescribing during the first trimester was associated with an increased risk of any major malformation (*v* penicillins; adjusted risk ratio 1.50, 95% confidence interval 1.13 to 1.99). Findings for clarithromycin had wide confidence intervals and analyses for azithromycin were precluded because of few events (supplementary table S8). Subgroup analyses of prescribing for less than one week were not informative because 94.7% of macrolide prescriptions were for 5-7 days (supplementary table S9). Because no correction for multiple testing was applied, we would expect three of the 62 subgroup tests to be statistically significant (at α=0.05) by chance alone.

### Sensitivity analyses

**Fig 4:**
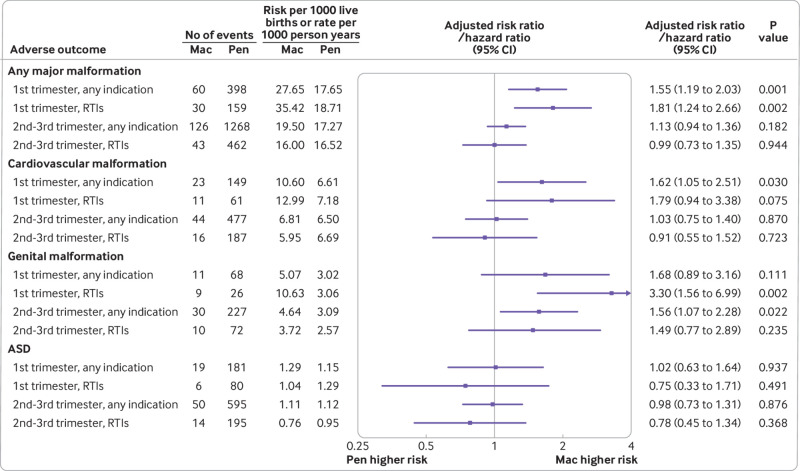
Association between adverse child outcomes and macrolide (*v* penicillin) prescribing during pregnancy for any indication (main analyses) and restricted to mothers in whom antibiotics were prescribed for respiratory tract infections by timing of prescription: during first trimester (from****four to****13 gestational weeks); and during second to third trimester (from 14 gestational weeks to delivery). To protect confidentiality of patient data and to be concise, outcomes are only given when all four analyses had at least five events in macrolides group. ASD=autism spectrum disorder; Mac=macrolide; Pen=penicillin; RTI=respiratory tract infection

We found no associations between adverse child outcomes and macrolides (*v* penicillins) prescribed before conception (fig 3). We found no associations between any major malformation, cardiovascular malformations, or genital malformations and having siblings who were prenatally exposed to macrolides versus penicillins; however, numbers were limited for many outcomes by trimester (supplementary table S10). Sensitivity analyses restricted to mothers who were prescribed macrolides or penicillins for respiratory tract infections during pregnancy did not change the findings of the primary analyses, although the analyses were underpowered for many outcomes (fig 4 and supplementary table S11). After we adjusted for potential bias owing to outcome misclassification and live birth bias, the estimated risk ratio for any major malformation of macrolide (*v* penicillin) prescribing during the first trimester increased to 1.58 (95% confidence interval 1.22 to 2.08); the estimated risk ratio for cardiovascular malformations increased to 1.78 (1.12 to 2.80; supplementary text S3).

Post hoc exploratory analyses of associations between macrolide prescribing during the first trimester and six common specific malformations were with limited power and revealed no statistically significant results. However, we found an increased risk of hypospadias and craniosynostosis in children of mothers prescribed macrolides during the second or third trimester (supplementary table S12).

## Discussion

Children of mothers prescribed macrolide antibiotics during the first trimester of pregnancy had an increased risk of any major malformation and specifically cardiovascular malformations compared with children of mothers prescribed penicillin antibiotics. These associations were not significant for prescriptions during the second or third trimester. The risk of genital malformations was increased in children of mothers prescribed macrolides compared with penicillins in any trimester, although associations were not statistically significant when prescribing was restricted to the first trimester. We found no statistically significant associations between macrolide prescribing and neurodevelopmental disorders. Assuming the associations are causal, we estimate that for every 1000 mothers prescribed macrolides instead of penicillins during the first trimester, an additional 4.1 (95% confidence interval 0.4 to 9.4) children would have cardiovascular malformations; the corresponding figures for prescriptions during any trimester and genital malformations would be 1.7 (0.4 to 3.5). Subgroup and sensitivity analyses did not change these findings.

### Strengths and weaknesses of study

The strengths of our study include the large, population based sample of mothers and children registered with primary care in the UK. The key challenge of using observational studies is to separate the potential adverse effects of antibiotic prescribing from the effects of infection on the fetus. We addressed this challenge by comparing the children of mothers prescribed macrolides with those whose mothers were prescribed penicillins because the indications for these treatments largely overlapped (supplementary table S13). We also restricted analyses to mothers prescribed one monotherapy of either macrolides or penicillins to reduce the risk of confounding because of severe or recurrent infections. We also performed sensitivity analyses that were restricted to mothers with respiratory tract infections, which are largely caused by virus infections, to minimise the treatment benefits of the antibiotics. We adjusted for measured confounders by using propensity score matching, and evaluated the effects of unmeasured maternal characteristics by using two negative control cohorts: children of mothers prescribed macrolides or penicillins before pregnancy; and siblings of children prenatally exposed to macrolides or penicillins in the study cohort.

The increased risk of any major malformation associated with macrolide prescribing in the first trimester, but not later in pregnancy, is consistent with the critical period of fetal organogenesis; that is, from five to 13 gestational weeks for most major malformations, including cardiovascular malformations. The increased risk of genital malformations (mainly hypospadias) persisted after the first trimester, which corresponds with evidence from animal studies that genitalia development could be susceptible to insults after early pregnancy.^30^ ^31^ ^32^ This specificity of exposure timing also points to adverse effects of macrolides rather than effects caused by unmeasured systematic differences between groups (eg, socioeconomic status or genetic factors).

A key weakness of the study is the limited power to examine treatment exposure during known critical periods for specific malformations and neurodevelopmental disorders, thereby inducing a potential dilution bias towards the null. To avoid numerous, underpowered comparisons, we grouped prescribing according to trimesters, not known critical periods (eg, 5-10 gestational weeks for cardiovascular malformations),^33^ and we categorised malformations by organ system. Jenkins and colleagues highlighted the possible effect dilution in epidemiological studies when phenotypes with different inherent susceptibilities are grouped together.^34^

A further limitation is that we analysed antibiotic prescribing as our main exposure, not dispensing or use, which were not recorded in the database. Additionally, compliance rates with erythromycin treatment could be lower than those for penicillin because of its gastrointestinal side effects. However, more untreated maternal infections in the macrolide group are unlikely to explain our findings because a sensitivity analysis restricted to respiratory tract infections showed similar results.^35^ We also quantified the potential underestimation of our findings because of outcome misclassification and live birth bias. Although unmeasured confounders exist, the negative control analyses based on prescribing before conception and outcomes in siblings did not change our results. Our algorithm to estimate the start date of pregnancy probably overestimated the duration of pregnancy in a small proportion of pregnancies (supplementary text S1).

### Comparison with other studies

A study that used the Swedish birth registry found a similar magnitude of effect between first trimester erythromycin prescribing (compared with all live born) and major cardiovascular malformations.^36^ However, most other studies reported no association between the use of macrolides during the first trimester or during the whole pregnancy and major malformations (supplementary table S14). One reason could be a dilution effect because of macrolide prescribing outside the period of organogenesis.^23^ This finding is especially of concern when a considerable proportion (more than one third in our study, 36%) of first trimester macrolides were prescribed very early in pregnancy (before four gestational weeks), probably before pregnancy had been detected in most cases.^36^ Another explanation is lack of power; five out of nine previous studies reported up to 15 malformations in macrolide groups.

### Implications

The potential mechanisms for the association between adverse child outcomes and prenatal macrolide treatment were discussed after findings from the ORACLE II trial were published.^37^ One of the pathways hypothesised was the arrhythmic effect of macrolides, which is thought to induce increased risks of cardiovascular events and mortality in high risk adults.^7^ ^8^ Experimentally, the arrhythmic effect of certain drugs can lead to fetal hypoxia, and might underlie the association between macrolide prescribing during pregnancy and increased risks of malformations.^13^ ^38^ Animal studies of clarithromycin and azithromycin reported a high risk of embryotoxicity (including fetal growth restriction and death) and teratogenicity that were dose dependent.^2^ ^14^ ^39^

We found no evidence of an association between neurodevelopmental disorders and macrolide prescribing during pregnancy, in contrast to previous studies.^37^ ^40^ Our finding of no association might be because of the multifactorial causes of neurodevelopmental disorders. For example, 70% of cerebral palsy cases are considered to have non-genetic causes, whereas 60-70% of epilepsy, attention deficit hyperactivity disorder, and autism spectrum disorder cases are attributable to genetic factors.^41^ ^42^ ^43^ ^44^ The critical period for cerebral palsy could also be restricted (eg, third trimester for spastic cerebral palsy).^45^ In the ORACLE II trial, an increased risk of cerebral palsy was found in children of mothers with spontaneous preterm labour, in whom erythromycin was prescribed at a median of 31 gestational weeks.^37^

The increased risks of major malformations found in this study and in the study by Källén and colleagues^36^ provide evidence that macrolide prescribing during pregnancy warrants caution. If the associations are causal, we estimate that an additional 4.1 (95% confidence interval 0.4 to 9.4) children with cardiovascular malformations and 1.7 (95% CI: 0.4 to 3.5) children with genital malformations would occur for every 1000 children exposed to macrolides instead of penicillins in the first trimester or in any trimester, respectively. Drug safety leaflets should report that there is uncertainty about the safety of macrolides, including erythromycin, and recommend use of alternative antibiotics when feasible until further research is available. Given the widespread use of macrolides during pregnancy, an urgent need exists for international collaboration. We need to bring together existing datasets for large scale analyses of high quality trials and observational cohorts that have accurate measurements of treatments and child outcomes. Analyses should prespecify treatment exposure periods based on gestational days when hazards are likely to impact on organogenesis for specific malformations or on neurodevelopmental outcomes.

### Conclusions

This population based analysis showed that prescribing macrolide antibiotics rather than penicillin antibiotics during the first trimester of pregnancy was associated with an increased risk of any major malformation and specifically cardiovascular malformation. We also found an increased risk of genital malformations associated with macrolide prescribing in any trimester. These findings call for cautious use of macrolides during pregnancy. Drug safety leaflets should report that there are concerns about the safety of macrolides, including erythromycin, and recommend the use of alternative antibiotics when feasible until further research is available.

**What is already known on this topic**

A recent systematic review on macrolide prescribing during pregnancy showed consistent evidence for increased risk of miscarriage but less consistent evidence for increased risk of congenital malformations, cerebral palsy, and epilepsy
Policy advice about use of macrolides during pregnancy varies across countries

**What this study adds**

Macrolide antibiotics prescribing during the first trimester of pregnancy was associated with an increased risk of any major malformation and specifically cardiovascular malformations compared with penicillin antibiotics
Macrolide antibiotics prescribing in any trimester was associated with an increased risk of genital malformations
Macrolide antibiotics should be used with caution during pregnancy and if feasible alternative antibiotics should be prescribed until further research is available

## Web extra

Extra material supplied by authors

**Web appendix:** Supplementary appendix

## Notes

Contributors: All authors were involved in the conception of the research question and designed the protocol. HF did the statistical analysis. HF, LL, and RG designed the tables, figures, and appendices. HF prepared the initial drafts of the manuscript, with input from LL, RG, and FOC. FOC validated the cerebral palsy cases. All authors critically reviewed and approved the final version of the manuscript. RG and LL are the guarantors. The corresponding author attests that all listed authors meet authorship criteria and that no others meeting the criteria have been omitted.

Ethical approval: This study was approved by the University College London Research Ethics Committee (14569.001).

Data sharing: No additional data available.

The lead author (HF) affirms that the manuscript is an honest, accurate, and transparent account of the study being reported; that no important aspects of the study have been omitted; and that any discrepancies from the study as registered have been explained.

Dissemination to participants and related patient and public communities: The results of the research will be disseminated to the public through broadcasts, popular science articles, and newspapers.
