# Associations between macrolide antibiotics prescribing during pregnancy and adverse child outcomes in the UK: population based cohort study

**DOI:** 10.1136/bmj.m766

**Published:** 2020-03-04

In this paper by Fan and colleagues (*BMJ* 2020;368:m331, https://doi.org/10.1136/bmj.m331, published 19 February 2020), the statement “The United Kingdom Medicines and Healthcare products Regulatory Agency advise that alternatives to clarithromycin and azithromycin should be prescribed during pregnancy” incorrectly implied that the Medicines and Healthcare products Regulatory Authority (MHRA) has issued advice on the relative use of macrolides during pregnancy. The Summaries of Product Characteristics, which are authorised by the MHRA, state that for clarithromycin, “use during pregnancy is not advised without carefully weighing the benefits against risk”; for azithromycin, “azithromycin should only be used during pregnancy if the benefit outweighs the risk”; and for erythromycin, “like all drugs erythromycin should be used in pregnancy only when clearly indicated” (see table 1 below). The authors acknowledge the support from CALIBER, led from the UCL Institute of Health Informatics. This study is based in part on data from the CPRD obtained under license. The interpretation and conclusions contained in this study are those of the authors alone.

**Table 1:** Statements from British National Formulary (BNF) and in Summaries of Product Characteristics (SPC) on the safety of specific macrolides during pregnancy

Drug	Statement
Erythromycin	
SPC	Erythromycin 250 mg gastroresistant tablets (https://mhraproductsprod.blob.core.windows.net/docs-20200210/bcaf2e97bcf6284e78173c7a70022a8049abbbcb): There are no adequate and well-controlled studies in pregnant women. However, observational studies in humans have reported cardiovascular malformations after exposure to medicinal products containing erythromycin during early pregnancy. Erythromycin has been reported to cross the placental barrier in humans, but foetal plasma levels are generally low. There have been reports that maternal macrolide antibiotics exposure within 7 weeks of delivery may be associated with a higher risk of infantile hypertrophic pyloric stenosis (IHPS). Erythromycin is excreted in breast milk. Caution should be exercised when administering erythromycin to lactating mothers due reports of infantile hypertrophic pyloric stenosis in breast-fed infants. Erythromycin 250mg gastroresistant hard capsules (https://mhraproductsprod.blob.core.windows.net/docs-20200210/8a6d7389440268b9a42f4de6d0b470e7de776b0e): There are no adequate and well-controlled studies in pregnant women. However, observational studies in humans have reported cardiovascular malformations after exposure to medicinal products containing erythromycin during early pregnancy. Like all drugs erythromycin should be used in pregnancy only when clearly indicated. Erythromycin crosses the placental barrier. Erythromycin has been reported to cross the placental barrier in humans, but foetal plasma levels are generally low. Nursing mothers: erythromycin is excreted in human milk and should be used in lactating women only if clearly needed.
BNF	Not known to be harmful (https://bnf.nice.org.uk/drug/erythromycin.html#pregnancy).
Clarithromycin	
SPC	Clarithromycin 250 mg film coated tablets (https://mhraproductsprod.blob.core.windows.net/docs-20200210/54bf0107e3046a6dc6a758d70e59120da384cfbe): The safety of clarithromycin for use during pregnancy has not been established. Based on variable results obtained from studies in mice, rats, rabbits and monkeys, the possibility of adverse effects on embryofoetal development cannot be excluded. Therefore, use during pregnancy is not advised without carefully weighing the benefits against risk. Clarithromycin 125 mg/5 mL suspension (https://mhraproductsprod.blob.core.windows.net/docs-20200210/28d6a0d4390cae3282846d9595a836f9ff495b68): Data on the use of clarithromycin during the first trimester of more than 200 pregnancies show no clear evidence of teratogenic effects, or of adverse effects on the health of the neonate. Data from a limited number of pregnant women exposed in the first trimester indicate a possible increased risk of abortions. To date no other relevant epidemiological data are available. Data from animal studies have shown reproductive toxicity (see section 5.3). The risk for humans is unknown. Clarithromycin should only be used during pregnancy after a careful benefit/risk assessment.
BNF	Manufacturer advises avoid, particularly in the first trimester, unless potential benefit outweighs risk (https://bnf.nice.org.uk/drug/clarithromycin.html#pregnancy).
Azithromycin	
SPC	Azithromycin 250 mg tablets (https://mhraproductsprod.blob.core.windows.net/docs-20200210/693e677325f9ec4ff7dd7362e521eaca1e167d87): There are no adequate data from the use of azithromycin in pregnant women. In reproduction toxicity studies in animals azithromycin was shown to pass the placenta, but no teratogenic effects were observed (see section 5.3). The safety of azithromycin has not been confirmed with regard to the use of the active substance during pregnancy. Therefore azithromycin should only be used during pregnancy if the benefit outweighs the risk.
BNF	Manufacturers advise use only if adequate alternatives not available (https://bnf.nice.org.uk/drug/azithromycin.html#pregnancy).

