# Supplementary material for: Associations between macrolide antibiotics prescribing during pregnancy and adverse child outcomes in the UK: population based cohort study
Source: BMJ. 2020 Feb 19;368:m331. doi: 10.1136/bmj.m331 (PMC7190043; doi:10.1136/bmj.m331)
Supplement: Supplementary file 1 — Web appendix: Supplementary appendix [file fanh050922.ww.pdf]

Supplementary Appendix

Heng Fan,[1] researcher, Ruth Gilbert,[1] professor of clinical epidemiology, Finbar O'Callaghan,[2] professor of paediatric neuroscience, Leah Li,[1] professor of medical statistics and epidemiology

[1]Population, Policy and Practice Programme, Great Ormond Street Institute of Child Health, University College London, London, UK.
[2]Developmental Neurosciences Programme, Great Ormond Street Institute of Child Health, University College London, London, UK.

Table S1. The RECORD statement. 2
Text S1. Development of gestational age in the Clinical Practice Research Datalink (CPRD). 8
Text S2. Outcome identification. 9
Table S2. Codes for outcome identification. 10
Table S3. Most frequent five Read codes for each system-specific malformation. 11
Table S4. Definition of covariates.* 13
Table S5. Unadjusted and propensity-score-adjusted baseline characteristics (N [%]) of children whose mother were prescribed macrolides or penicillins from 14 gestation weeks to delivery (“the second to third trimester”). 16
Table S6. Unadjusted and propensity-score-adjusted baseline characteristics (N [%]) of children whose mother were prescribed macrolides or penicillins from 4 gestation weeks to delivery (“in any trimester”). 17
Table S7. Unadjusted and propensity-score-adjusted baseline characteristics (N [%]) of children whose mother were prescribed macrolides or penicillins 10 to 50 weeks before pregnancy. 18
Table S8. Subgroup analysis according to macrolides subtypes, on the association between adverse child outcomes and macrolides versus penicillins prescribed during pregnancy. 19
Table S9. Subgroup analysis according to duration of treatment (< 7 days or ≥ 7 days), on the association between adverse child outcomes and macrolides versus penicillins prescribed during pregnancy. 20
Table S10. Sensitivity analysis: comparison of the risks (or hazards) between siblings of children prenatally prescribed macrolides and siblings of children prenatally prescribed penicillins in the study cohort, according to timing of prescription. 22
Table S11. Sensitivity analysis on the association between adverse child outcomes and macrolides versus penicillins prescribed during pregnancy: restricting to mothers whose antibiotics were prescribed to respiratory tract infections. 23
Text S3. Probabilistic multiple bias analysis on outcome misclassification and live- birth bias 24
Table S12. *Post-hoc* analysis on the association between common specific malformation and macrolides versus penicillins prescribed during pregnancy. 29
Table S13. Number of prescriptions matched or not matched with any indication (infection) and number of any major malformation by each indication. 30
Table S14. Previously published studies on the association between maternal exposure of macrolides and major congenital malformations or neurodevelopmental disorders. 31
Reference 33

Table S1. The RECORD statement.

	Item No.	STROBE items	Location in manuscript where items are reported	RECORD items	Location in manuscript where items are reported
**Title and abstract**					
	1	(a) Indicate the study's design with a commonly used term in the title or the abstract (b) Provide in the abstract an informative and balanced summary of what was done and what was found	1-2	RECORD 1.1: The type of data used should be specified in the title or abstract. When possible, the name of the databases used should be included.<br><br>RECORD 1.2: If applicable, the geographic region and timeframe within which the study took place should be reported in the title or abstract.<br><br>RECORD 1.3: If linkage between databases was conducted for the study, this should be clearly stated in the title or abstract.	1<br><br>2<br><br>-
**Introduction**					
Background rationale	2	Explain the scientific background and rationale for the investigation being reported	4		
Objectives	3	State specific objectives, including any prespecified hypotheses	4		
**Methods**					
Study Design	4	Present key elements of study design early in the paper	5		
Setting	5	Describe the setting, locations, and relevant dates, including periods of recruitment, exposure, follow-up, and data collection	5		

Participants	6	*(a) Cohort study* - Give the eligibility criteria, and the sources and methods of selection of participants. Describe methods of follow-up<br>*Case-control study* - Give the eligibility criteria, and the sources and methods of case ascertainment and control selection. Give the rationale for the choice of cases and controls<br>*Cross-sectional study* - Give the eligibility criteria, and the sources and methods of selection of participants<br><br>*(b) Cohort study* - For matched studies, give matching criteria and number of exposed and unexposed<br>*Case-control study* - For matched studies, give matching criteria and the number of controls per case	5	RECORD 6.1: The methods of study population selection (such as codes or algorithms used to identify subjects) should be listed in detail. If this is not possible, an explanation should be provided.<br><br>RECORD 6.2: Any validation studies of the codes or algorithms used to select the population should be referenced. If validation was conducted for this study and not published elsewhere, detailed methods and results should be provided.<br><br>RECORD 6.3: If the study involved linkage of databases, consider use of a flow diagram or other graphical display to demonstrate the data linkage process, including the number of individuals with linked data at each stage.	5<br><br>5<br><br>-
Variables	7	Clearly define all outcomes, exposures, predictors, potential confounders, and effect modifiers. Give diagnostic criteria, if applicable.	6-7	RECORD 7.1: A complete list of codes and algorithms used to classify exposures, outcomes, confounders, and effect modifiers should be provided. If these cannot be reported, an explanation should be provided.	Supplementary Text S1-2, table S2,Table S2
Data sources/ measurement	8	For each variable of interest, give sources of data and details of methods of assessment (measurement).<br>Describe comparability of assessment methods if there is more than one group	7		
Bias	9	Describe any efforts to address potential sources of bias	6,8-9		

Study size	10	Explain how the study size was arrived at	6, Supplementary Text S2		
Quantitative variables	11	Explain how quantitative variables were handled in the analyses. If applicable, describe which groupings were chosen, and why	Supplementary Table S4		
Statistical methods	12	(a) Describe all statistical methods, including those used to control for confounding<br>(b) Describe any methods used to examine subgroups and interactions<br>(c) Explain how missing data were addressed<br>(d) *Cohort study* - If applicable, explain how loss to follow-up was addressed<br>*Case-control study* - If applicable, explain how matching of cases and controls was addressed<br>*Cross-sectional study* - If applicable, describe analytical methods taking account of sampling strategy<br>(e) Describe any sensitivity analyses	7-8		
Data access and cleaning methods		..		RECORD 12.1: Authors should describe the extent to which the investigators had access to the database population used to create the study population.<br><br>RECORD 12.2: Authors should provide information on the data cleaning methods used in the study.	5<br><br>Figure 1
Linkage		..		RECORD 12.3: State whether the study included person-level, institutional-level, or	-

				other data linkage across two or more databases. The methods of linkage and methods of linkage quality evaluation should be provided.	
**Results**					
Participants	13	(a) Report the numbers of individuals at each stage of the study (*e.g.*, numbers potentially eligible, examined for eligibility, confirmed eligible, included in the study, completing follow-up, and analysed)<br>(b) Give reasons for non-participation at each stage.<br>(c) Consider use of a flow diagram	9-10<br>Figure 1<br>Figure 1	RECORD 13.1: Describe in detail the selection of the persons included in the study (*i.e.,* study population selection) including filtering based on data quality, data availability and linkage. The selection of included persons can be described in the text and/or by means of the study flow diagram.	Figure 1
Descriptive data	14	(a) Give characteristics of study participants (*e.g.*, demographic, clinical, social) and information on exposures and potential confounders<br>(b) Indicate the number of participants with missing data for each variable of interest<br>(c) *Cohort study* - summarise follow-up time (*e.g.*, average and total amount)	Table 1<br>Table 1<br>9-10		
Outcome data	15	*Cohort study* - Report numbers of outcome events or summary measures over time<br>*Case-control study* - Report numbers in each exposure category, or summary measures of exposure<br>*Cross-sectional study* - Report numbers of outcome events or summary measures	Table 2		
Main results	16	(a) Give unadjusted estimates and, if applicable, confounder-adjusted estimates and their precision (e.g., 95% confidence interval). Make clear which confounders	Table 2, 10-11		

		were adjusted for and why they were included<br>(b) Report category boundaries when continuous variables were categorized<br>(c) If relevant, consider translating estimates of relative risk into absolute risk for a meaningful time period	Supplementary Table S4<br><br>13		
Other analyses	17	Report other analyses done—e.g., analyses of subgroups and interactions, and sensitivity analyses	11		
**Discussion**					
Key results	18	Summarise key results with reference to study objectives	13		
Limitations	19	Discuss limitations of the study, taking into account sources of potential bias or imprecision. Discuss both direction and magnitude of any potential bias	13-14	RECORD 19.1: Discuss the implications of using data that were not created or collected to answer the specific research question(s). Include discussion of misclassification bias, unmeasured confounding, missing data, and changing eligibility over time, as they pertain to the study being reported.	13-14
Interpretation	20	Give a cautious overall interpretation of results considering objectives, limitations, multiplicity of analyses, results from similar studies, and other relevant evidence	15-16		
Generalisability	21	Discuss the generalisability (external validity) of the study results	13		
**Other Information**					
Funding	22	Give the source of funding and the role of the funders for the present study and, if applicable, for the original study on which the present article is based	17		
Accessibility of protocol, raw		..		RECORD 22.1: Authors should provide information on how to access any	Protocol: 6<br>Raw data:18

data, and programming code				supplemental information such as the study protocol, raw data, or programming code.	

*Reference: Benchimol EI, Smeeth L, Guttmann A, Harron K, Moher D, Petersen I, Sørensen HT, von Elm E, Langan SM, the RECORD Working Committee. The REporting of studies Conducted using Observational Routinely-collected health Data (RECORD) Statement. *PLoS Medicine* 2015; in press.

*Checklist is protected under Creative Commons Attribution (CC BY) license.

Text S1. Development of gestational age in the Clinical Practice Research Datalink (CPRD).

A hierarchy of available pregnancy markers was chosen that reflects their potential accuracy to estimate the start of a pregnancy episode. Pregnancy markers that directly provide gestational age such as gestational age in weeks, prenatal examination, and fertility procedures (IVF) were on the top of the hierarchy. Next hierarchy of markers includes ranges of gestational week indicators (e.g. premature 24-26 weeks) and outcome-specific estimates (e.g. premature labour, imputed as 36 weeks, because around 60% live premature births born at 36 gestational weeks). Gestational weeks imputed from birthweight was on the 3rd hierarchy, based on the intrauterine growth curves published by Irene E. Olsen et al.(1) For pregnancies with no information available for the above three hierarchies of markers, full term births were assumed and gestational week 40 were used to calculate pregnancy start dates. Codes used in each hierarchy is referenced from Matcho, A. et al. (2)

For babies in the study population (n=726274), gestational ages were measured from each hierarchy with the following proportion: 27.8% from the first hierarchy (from codes for gestational age), 14.3% from the second hierarchy (from codes for gestational week range), 8.2% from the third hierarchy (imputed based on birthweight), 49.6% imputed as full-term (40 gestational weeks). The distribution of gestational age is consistent with the UK Office of National Statistics, although about 6%-7% full-term births with "true" gestational age of 37-38 weeks might have been estimated to be with 39 or longer gestational weeks (see table below).(3) This equates to move the measurement window forward about two week earlier (from gestational week 2 instead of 4) for these live births, which would mildly bias the current association for the first trimester towards the null. Similar algorithms (using hierarchical code groups and imputing to estimate pregnancy start dates) were reported and validated in other studies of CPRD, showing close agreement with external data.(2, 4)

TextS1.Table-1 Distribution of gestational weeks of live births in the target population of this study and according to the UK Office of National Statistics.

Gestational age, weeks	This study 1990-2015		The UK Office for National Statistics 2007-08
	No. of live births	Proportion	
23-27	3848	0.5%	0.5%
28-31	6271	0.9%	0.8%
32-34	11835	1.6%	1.9%
35-36	36386	5.0%	4.1%
37-38	87666	12.1%	19.3%
>=39	580268	79.9%	73.5%

Text S2. Outcome identification.

The main outcomes of this study were major (any and five system-specific) malformations and four neurodevelopmental disorders.

Eligible outcomes for this study include those could potentially result from short-term fetal hypoxia. We therefore included major malformations (any and system-specific malformations) and neurodevelopmental disorders. Malformations with specific known causes such as malformation resulted from maternal infections, fetal alcohol syndrome, Valproate syndrome and chromosomal malformations were not included. Twelve system-specific malformations were defined according to the European Surveillance of Congenital Anomalies (EUROCAT).(5)

We then excluded 1) the musculoskeletal malformation (e.g. club foot, knock-knee and hip dislocation) as a system-specific malformation and as "any major malformation", because they are not reliably recorded in GP records (6); and 2) system-specific malformations that we had insufficient power to detect a 2-fold relative risk increase at 80% power (5% α level). Five out of the eleven system-specific malformations from the EUROCAT classification fulfilled the power criterion according to its prevalence table and were analysed as system-specific malformations, including nervous system malformation, cardiovascular malformation, gastrointestinal malformation, genital malformation and urinary malformation (details were described in our protocol on www.clinicaltrials.gov [NCT03948620]).(7)

Any of the eleven system-specific malformations (except for musculoskeletal malformations) was evaluated as "any major malformation", and identified from child GP records by 3 years old using Read codes which were mapped to the tenth edition of the International Classification of Diseases (ICD–10) code lists provided by EUROCAT.(5)

Neurodevelopmental disorders (cerebral palsy, epilepsy, ADHD and ASD) were defined as the time to the first diagnostic or treatment code indicating the outcome by 14 years old. We identified potential cerebral palsy cases based on informative prescription or Read codes using the Random Forest approach, as we have previously described.(8) The potential cerebral palsy cases were then validated by a paediatric-neurologist (FC) blinded to the prenatal antibiotics exposure. Other neurodevelopmental disorders (epilepsy, ADHD and ASD) were identified using previously validated criteria using diagnostic codes and/or prescriptions (Supplementary Table S2).(9-11)

Table S2. Codes for outcome identification.

Outcome	Case identification
Any major congenital malformation	Any major system specific malformation according to the EUROCAT classification. We use Read code lists mapped to ICD 10 codes Chapter Q. Exclude: 1) minor anomalies post-2005*; 2) malformations caused by known chromosomal abnormalities and teratogens (i.e. Teratogenic syndromes with malformations, Fetal alcohol syndrome, Valproate syndrome, Maternal infections resulting in malformations, Genetic syndromes + microdeletions, Chromosomal malformations); and 3) musculoskeletal malformations.
Cardiovascular	Read codes mapped to ICD 10 (Q20-Q26, exclude Q2111, Q250 if GA <37 weeks, Q2541, Q256 if GA<37 weeks, Q261)
Gastrointestinal	Read codes mapped to ICD 10 (Q38-Q45, Q790, exclude Q381, Q382, Q3850, Q400, Q401, Q4021, Q430, Q4320, Q4381, Q4382)
Nervous system	Read codes mapped to ICD 10 (Q00-Q07, exclude Q0461, Q0782)
Genital	Read codes mapped to ICD 10 (Q50-Q52, Q54-Q56, exclude Q523, Q525, Q527, Q5520, Q5521)
Urinary	Read codes mapped to ICD 10 (Q60-Q64, Q794, exclude Q610, Q627, Q633)
Cerebral palsy	Besides cases identified by ≥ 1 diagnostic code, we identified cerebral palsy cases from informative prescription or Read codes using the Random Forest approach and were validated by a paediatric-neurologist (FC) blinded to the prenatal antibiotics exposure.(8)
Epilepsy	Two prescriptions of antiepileptic drug (AED, identified based on British National Formula Chapter 4.8) within four months or ≥ 1 diagnosis (11)
Attention deficit hyperactivity disorder (ADHD)	≥ 2 occurrence of prescriptions for ADHD (identified based on British National Formula Chapter 4.4) or diagnoses (attention deficit hyperactivity disorder, hyperkinetic disorders, hyperkinetic syndrome, hyperkinetic reaction of childhood or adolescence, overactive child syndrome and disturbance of activity and attention) within 4 month (9)
Autism spectrum disorder (ASD)	At least one diagnostic code ((infantile or childhood) autism, Asperger's syndrome, Rett's syndrome, Heller's syndrome, Autistic spectrum disorder, disintegrative disorder, and other pervasive developmental disorders) (10)

*The mapping from ICD 10 code to Read code was performed using R package "CALIBERcodelists". EUROCAT revised its list of minor anomalies at 2005, and we applied the updated "Excluded minor anomalies post-2005" list in this study. GA: gestational age.

Table S3. Most frequent five Read codes for each system-specific malformation.

Type	Description	Read code	ICD10 Code	Frequency
Cardiovascular	Ventricular septal defect	P54..00	Q210	382
	Patent ductus arteriosus	P70..00	Q250	189
	Atrial septal defect NOS	P550.00	Q211	116
	Ostium secundum atrial septal defect	P71..00	Q211	35
	Coarctation of aorta	P55..00	Q251	33
Genital	Hypospadias	PC60.00	Q54	293
	Hypospadias, glandular	PC60312	Q540	20
	Hypospadias, penile	PC60000	Q541	14
	Hypospadias, glanular	PC60311	Q540	10
	Hooded penis	PCyy000	Q54	10
Neurological	Microcephalus	P21..00	Q02	44
	Spina bifida	P1...00	Q05	26
	Congenital hydrocephalus	P23..00	Q03	12
	Septo-optic dysplasia	P246.00	Q044	6
	Micrencephaly	P211.00	Q02	6
Eye	Congenital ptosis	P360.00	Q100	46
	Congenital cataract, unspecified	P330.00	Q120	14
	Coloboma of iris	P344200	Q130	14
	Congenital cataract and lens anomalies	P33..00	Q12	12
	Congenital lacrimal passage anomalies	P364.00	Q106	10
Orofacial cleft	Cleft palate	P90..00	Q35	38
	Cleft palate with cleft lip	P92..00	Q37	37
	Repair of cleft palate	7525.12	Q35	34
	Repair of cleft lip operations	7502.11	Q36	34
	Primary repair of cleft palate, unspecified	7525000	Q35	27
Urinary	Congenital hydronephrosis	PD23.00	Q620	30
	Multicystic kidney	PD13.11	Q611-Q614	19
	Congenital absence of kidney	PD02.00	Q600-Q602	15
	Dysplasia of kidney	PD04.00	Q614	14
	Horseshoe kidney	PD38.00	Q631	10
Gastrointestinal	Hirschsprung's disease	PB30.00	Q431	27
	Imperforate anus	PB26.00	Q423	17
	Atresia of oesophagus	PA30.00	Q39	11
	Other anomalies of lip	PA2A.00	Q380	9
	Atresia of duodenum	PB10100	Q410	6
Respiratory	Choanal atresia	P80..00	Q300	7
	Other lung anomalies	P86..00	Q338	6
	Congenital cystic lung	P84..00	Q330	5
	Congenital bronchomalacia	P83yB00	Q322	<5
	Congenital bronchogenic cyst	P843.12	Q330	<5
Ear & face	Ear anomalies with hearing impairment	P40..00	Q169	8
	Eustachian tube anomalies	P423.00	Q164	<5
	Other specified face and neck anomalies	P4y..00	Q188	<5
	Absence of ear NOS	P401011	Q160	<5
	Deafness due to congenital anomaly NEC	P40z.11	Q169	<5
Abdominal wall defects	Gastroschisis	PG71.00	Q793	19
	Exomphalos	PG70.00	Q792	<5

	Abdominal wall anomalies	PG7..00	Q795	<5
Other	Craniosynostosis	PG03.00	Q750	29
	Urticaria pigmentosa	PH32100	Q822	19
	Ichthyosis congenita	PH1..00	Q80	14
	Imperfect fusion of skull	PG06.00	Q750	12
	Scaphocephaly	PG03.11	Q750	8

*The frequencies were calculated for Read codes (not diagnosis) without de-duplication. In accordance with the confidentiality preserving policy of CPRD, we suppressed the information where the frequency cell contains <5 events (noted as "<5").

Table S4. Definition of covariates.*

Covariates	Time for measurement	Value	Description
Age at delivery	-	Grouped into categories of 5 calendar years (roughly): 14-19; 20-24; 25-29; 30-34; 35-50.	Defined as the calendar year of delivery minus mothers' year of birth.
Calendar year of delivery	-	Grouped into categories of 5 calendar years: 1990-1994; 1995-1999; 2000-2004; 2005-2009; 2010-2016.	-
Parity	-	Categorised as "0", and "≥ 1"	Number of times that the women has given live-birth which were captured in the CPRD Mother Baby Link before the current pregnancy.
Multiple births	-	"Singleton", and "(One of the) Twin, triplets, or quadruplets captured in the database".	
Alcohol misuse	Most recent measurement from 10 years before pregnancy to the end of pregnancy.	"Yes" and "No"	Alcohol misuse was defined as ≥ 14 units of alcohol per week, including moderate or severe drinker. Self-reported alcohol consumption was collected prospectively and coded by general practitioners or practice nurses on the consultation date in CPRD. The most recent alcohol consumption record was used to classify participants drinking behaviour, and "ex-drinker" was categorised as not alcohol misuser if there was no evidence of alcohol withdraw before pregnancy start. Alcohol misuse was defined using:<br>1) One of the Read codes indicating alcohol consumption; or,<br>2) A prescription for disulfarim or acamprosate; or,<br>3) Self-reported average weekly alcohol intakes >= 14 units in the "Additional Clinical Details".<br>We applied the code list of alcohol consumption developed by Bell at al.(12)
Illicit drug use	Most recent measurement from 10 years before pregnancy to the end of pregnancy.	"Yes" and "No"	Illicit drug use was defined using:<br>1) One of the Read codes indicating drug use, addiction, and overdose; or<br>2) A prescription for methadone treatment.<br>We assume that although a mother may stop using illicit drugs, the underlying behaviour was unlikely to vary significantly over time.
Obesity	Most recent measurement from 3 years before pregnancy till the end of the first trimester.	"Yes" and "No"	Mothers who were obese prior to the 2nd trimester of pregnancy were identified from the Read codes for obesity (or a BMI of ≥30 kg/m^2 - either directly entered or calculated from the most recent height measurement and median pre-pregnancy weight after excluding outliers. I.e. height outside the range 1-2m and weight outside the range 35-300kg, were removed). It was assumed that once a mother reached clinical obesity, the chance of her returning to a normal BMI in three years was minimal.

Tobacco use	Most recent measurement from 3 years before pregnancy to the end of pregnancy.	“Yes” and “No”	Tobacco use was defined as daily cigarette consumption of 1-100 cigarettes per day or other tobacco use. The most recent tobacco consumption record was used to classify participants drinking behaviour, and “ex-smoker” was categorised as non-recent smoker. Tobacco use was defined using:<br>1) One of the Read codes indicating tobacco consumption; or,<br>2) A prescription for smoking cessation aid; or,<br>3) Self-reported daily cigarette consumption of 1-100 cigarettes per day in the “Additional Clinical Details”.
Hypertension	50 weeks prior to delivery	“Yes” and “No”	Mothers with hypertension during pregnancy were identified based on<br>1) Systolic and diastolic blood pressure was above 140mmHg and 90mmHg, respectively, or,<br>2) One of the Read code for hypertension and associated diagnoses (including pre-eclampsia, eclampsia and HELLP syndrome), or,<br>3) One prescription for hypertension drugs from sections 2.2 and 2.5 of the BNF. This variable identified mothers with both treated and untreated hypertension in pregnancy.
Diabetes	50 weeks prior to delivery	“Yes” and “No”	Mothers with diabetes during pregnancy were identified based on:<br>1) One of the Read codes for type I, type II, or gestational diabetes; or<br>2) Two or more prescriptions for anti-diabetic medication; or<br>3) One of laboratory tests indicating diabetes (defined as ≥2 abnormal glucose tests, fasting glucose >7.0 millimoles per litre [mmol/L] or >126 milligrams per decilitre [mg/dL], plasma glucose after glucose tolerance test >11.1 mmol/L or 200mg/dL, glycated haemoglobin ≥ 6.5%, or within diabetes annual review) recorded in the “Additional Clinical Details”.
Epilepsy	50 weeks prior to delivery	“Yes” and “No”	≥ 2 prescriptions of antiepileptic drugs (AEDs) within 4 months or ≥ 1 diagnosis
Depression	50 weeks prior to delivery	“Yes” and “No”	≥ 2 occurrences of diagnostic code, treatment code or symptom
Anxiety	50 weeks prior to delivery	“Yes” and “No”	≥ 2 occurrences of diagnostic code, treatment code or symptom
Treatment of chronic medical conditions during pregnancy	During pregnancy	“Yes” and “No”	Existence of chronic medical conditions are defined as conditions that are sufficiently severe to require on-going treatment during pregnancy. Mothers were considered to have a chronic medical condition if they were issued ≥ 2 prescriptions (on separate days during pregnancy and not more than four months apart) for drugs from the same BNF section or paragraph. Drugs used to treat common conditions in pregnancy, including reflux (BNF section 1.2), nausea and vomiting (BNF section 4.6), and constipation (BNF section 1.3), were not included.

Genitourinary tract infection	During pregnancy	“Yes” and “No”	Common terms categorised as “Genitourinary tract infection” include urinary tract infection, cystitis, vaginitis and the prescription of Nitrofurantoin.
Sexually Transmitted Infection	During pregnancy	“Yes” and “No”	Common terms categorised as “Sexually Transmitted Infection” include chlamydia infection, trachoma, “TORCH” (Toxoplasmosis, Other agents such as HIV, Rubella, Cytomegalovirus and Herpes simplex) and other sexually transmitted infections (STIs).

*When the key codes indicating a binary condition were not identified in the medical history of a subject, we classified the subject as absence of the condition. There were no missing for multi-categorical covariates in this study (“Age at delivery” and “Calendar year of delivery”).

Table S5. Unadjusted and propensity-score-adjusted baseline characteristics (N [%]) of children whose mother were prescribed macrolides or penicillins from 14 gestation weeks to delivery ("the second to third trimester").

Characteristic	Unadjusted			Propensity-score-adjusted*		
	Macrolides	Penicillins	St.diff	Macrolides	Penicillins	St.diff
Number of children	6462	73429		6462	73400	
**Maternal baseline characteristic**						
Age at delivery			0.08			0.004
13-19	232 (3.6)	2889 (3.9)		232 (3.6)	2631.5 (3.6)	
20-24	825 (12.8)	10560 (14.4)		825 (12.8)	9273.3 (12.6)	
25-29	1562 (24.2)	19105 (26.0)		1562 (24.2)	17779.2 (24.2)	
30-34	2165 (33.5)	23514 (32.0)		2165 (33.5)	24589.0 (33.5)	
35-50	1678 (26.0)	17361 (23.6)		1678 (26.0)	19127.0 (26.1)	
Calendar year of delivery			0.054			0.003
1990-1994	606 (9.4)	7594 (10.3)		606 (9.4)	6907.4 (9.4)	
1995-1999	1067 (16.5)	13023 (17.7)		1067 (16.5)	12111.0 (16.5)	
2000-2004	1344 (20.8)	15025 (20.5)		1344 (20.8)	15234.9 (20.8)	
2005-2009	1688 (26.1)	18005 (24.5)		1688 (26.1)	19248.1 (26.2)	
2010-2016	1757 (27.2)	19782 (26.9)		1757 (27.2)	19898.6 (27.1)	
Alcohol misuse	308 (4.8)	3526 (4.8)	0.002	308 (4.8)	3494.4 (4.8)	<0.001
Illicit drug use	81 (1.3)	739 (1.0)	0.023	81 (1.3)	911.0 (1.2)	0.001
Tobacco use	2136 (33.1)	23351 (31.8)	0.027	2136 (33.1)	24190.8 (33.0)	0.002
Obesity	795 (12.3)	8046 (11.0)	0.042	795 (12.3)	8979.4 (12.2)	0.002
Hypertension	507 (7.8)	5355 (7.3)	0.021	507 (7.8)	5765.8 (7.9)	<0.001
Diabetes	254 (3.9)	2359 (3.2)	0.039	254 (3.9)	2868.7 (3.9)	0.001
Anxiety	187 (2.9)	1820 (2.5)	0.026	187 (2.9)	2118.0 (2.9)	<0.001
Depression	714 (11.0)	6891 (9.4)	0.055	714 (11.0)	8033.0 (10.9)	0.003
Epilepsy	35 (0.5)	474 (0.6)	0.014	35 (0.5)	429.1 (0.6)	0.006
**Pregnancy related characteristic**						
Parity ≥1	2367 (36.6)	26444 (36.0)	0.013	2367 (36.6)	26964.5 (36.7)	0.002
Multiple births	182 (2.8)	2018 (2.7)	0.004	182 (2.8)	2071.6 (2.8)	<0.001
Genitourinary tract infection	271 (4.2)	8725 (11.9)	0.286	271 (4.2)	3112.9 (4.2)	0.002
Sexually Transmitted Infection	179 (2.8)	936 (1.3)	0.106	179 (2.8)	2079.6 (2.8)	0.004
Treatment of chronic medical conditions	1328 (20.6)	12718 (17.3)	0.083	1328 (20.6)	14976.0 (20.4)	0.004

*Exposure propensity scores were measured as the predicted probability of receiving macrolides versus penicillins, conditional on the maternal and pregnancy related characteristics included in this table. 50 Strata were created based on the distribution of the propensity score of macrolides group. Weights for the penicillins group were calculated according to the distribution of the macrolides group among the strata and were used to estimate adjusted baseline characteristics. A meaningful between-group imbalance was assessed by an absolute standardised difference (Std.diff, the difference in means in units of standard deviation) of more than 0.1. Numbers in adjusted penicillins group were non-integer, because they were weighted based on the distribution of propensity score of macrolides group.

Table S6. Unadjusted and propensity-score-adjusted baseline characteristics (N [%]) of children whose mother were prescribed macrolides or penicillins from 4 gestation weeks to delivery ("in any trimester").

Characteristic	Unadjusted			Propensity-score-adjusted*		
	Macrolides	Penicillins	St.diff	Macrolides	Penicillins	St.diff
Number of children	8632	95973		8632	95971	
**Maternal baseline characteristic**						
Age at delivery			0.063			0.003
13-19	362 (4.2)	3875 (4.0)		362 (4.2)	3992.9 (4.2)	
20-24	1202 (13.9)	14070 (14.7)		1202 (13.9)	13291.0 (13.8)	
25-29	2086 (24.2)	25328 (26.4)		2086 (24.2)	23169.9 (24.1)	
30-34	2829 (32.8)	30406 (31.7)		2829 (32.8)	31559.9 (32.9)	
35-50	2153 (24.9)	22294 (23.2)		2153 (24.9)	23957.2 (25.0)	
Calendar year of delivery			0.066			0.003
1990-1994	776 (9.0)	9819 (10.2)		776 (9.0)	8662.3 (9.0)	
1995-1999	1385 (16.0)	16769 (17.5)		1385 (16.0)	15461.5 (16.1)	
2000-2004	1840 (21.3)	19429 (20.2)		1840 (21.3)	20448.7 (21.3)	
2005-2009	2256 (26.1)	23599 (24.6)		2256 (26.1)	25073.7 (26.1)	
2010-2016	2375 (27.5)	26357 (27.5)		2375 (27.5)	26324.9 (27.4)	
Alcohol misuse	437 (5.1)	4573 (4.8)	0.014	437 (5.1)	4823.4 (5.0)	0.002
Illicit drug use	112 (1.3)	982 (1.0)	0.026	112 (1.3)	1193.9 (1.2)	0.005
Tobacco use	2926 (33.9)	30763 (32.1)	0.039	2926 (33.9)	32235.1 (33.6)	0.007
Obesity	1057 (12.2)	10624 (11.1)	0.037	1057 (12.2)	11688.6 (12.2)	0.002
Hypertension	668 (7.7)	6978 (7.3)	0.018	668 (7.7)	7379.0 (7.7)	0.002
Diabetes	322 (3.7)	3141 (3.3)	0.025	322 (3.7)	3551.3 (3.7)	0.002
Anxiety	261 (3.0)	2376 (2.5)	0.034	261 (3.0)	2841.3 (3.0)	0.004
Depression	941 (10.9)	9179 (9.6)	0.044	941 (10.9)	10393.5 (10.8)	0.002
Epilepsy	60 (0.7)	629 (0.7)	0.005	60 (0.7)	666.4 (0.7)	<0.001
**Pregnancy related characteristic**						
Parity >=1	3149 (36.5)	34524 (36.0)	0.011	3149 (36.5)	35080.6 (36.6)	0.002
Multiple births	234 (2.7)	2553 (2.7)	0.003	234 (2.7)	2594.6 (2.7)	<0.001
Genitourinary tract infection	361 (4.2)	11521 (12.0)	0.29	361 (4.2)	3964.7 (4.1)	0.003
Sexually Transmitted Infection	281 (3.3)	1237 (1.3)	0.132	281 (3.3)	3075.3 (3.2)	0.003
Treatment of chronic medical conditions	1750 (20.3)	16784 (17.5)	0.071	1750 (20.3)	19480.0 (20.3)	0.001

*Exposure propensity scores were measured as the predicted probability of receiving macrolides versus penicillins, conditional on the maternal and pregnancy related characteristics included in this table. 50 Strata were created based on the distribution of the propensity score of macrolides group. Weights for the penicillins group were calculated according to the distribution of the macrolides group among the strata and were used to estimate adjusted baseline characteristics. A meaningful between-group imbalance was assessed by an absolute standardised difference (St.diff, the difference in means in units of standard deviation) of more than 0.1. Numbers in adjusted penicillins group were non-integer, because they were weighted based on the distribution of propensity score of macrolides group.

Table S7. Unadjusted and propensity-score-adjusted baseline characteristics (N [%]) of children whose mother were prescribed macrolides or penicillins 10 to 50 weeks before pregnancy.

Characteristic	Unadjusted			Propensity-score-adjusted*		
	Macrolides	Penicillins	St.diff	Macrolides	Penicillins	St.diff
Number of children	11874	70440		11874	70425.1	
**Maternal baseline characteristic**						
Age at delivery			0.028			0.003
13-19	499 (4.2)	3150 (4.5)		499 (4.2)	2975.8 (4.2)	
20-24	1706 (14.4)	10482 (14.9)		1706 (14.4)	10091.3 (14.3)	
25-29	3099 (26.1)	18495 (26.3)		3099 (26.1)	18437.4 (26.2)	
30-34	3760 (31.7)	22346 (31.7)		3760 (31.7)	22240.1 (31.6)	
35-50	2810 (23.7)	15967 (22.7)		2810 (23.7)	16680.6 (23.7)	
Calendar year of delivery			0.038			0.003
1990-1994	1034 (8.7)	6060 (8.6)		1034 (8.7)	6109.9 (8.7)	
1995-1999	1986 (16.7)	12376 (17.6)		1986 (16.7)	11827.1 (16.8)	
2000-2004	2451 (20.6)	14977 (21.3)		2451 (20.6)	14593.5 (20.7)	
2005-2009	3030 (25.5)	18099 (25.7)		3030 (25.5)	17960.3 (25.5)	
2010-2016	3373 (28.4)	18928 (26.9)		3373 (28.4)	19934.4 (28.3)	
Alcohol misuse	607 (5.1)	3248 (4.6)	0.023	607 (5.1)	3584.8 (5.1)	0.001
Illicit drug use	144 (1.2)	695 (1.0)	0.022	144 (1.2)	852.0 (1.2)	<0.001
Tobacco use	3991 (33.6)	22730 (32.3)	0.029	3991 (33.6)	23798.6 (33.8)	0.004
Obesity	1448 (12.2)	8015 (11.4)	0.025	1448 (12.2)	8605.3 (12.2)	0.001
Hypertension	899 (7.6)	5107 (7.3)	0.012	899 (7.6)	5328.1 (7.6)	<0.001
Diabetes	395 (3.3)	2339 (3.3)	<0.001	395 (3.3)	2355.4 (3.3)	0.001
Anxiety	311 (2.6)	1681 (2.4)	0.015	311 (2.6)	1846.0 (2.6)	<0.001
Depression	1180 (9.9)	6601 (9.4)	0.019	1180 (9.9)	7001.3 (9.9)	<0.001
Epilepsy	73 (0.6)	432 (0.6)	<0.001	73 (0.6)	437.7 (0.6)	0.001
**Pregnancy related characteristic**						
Parity ≥1	4197 (35.3)	26876 (38.2)	0.058	4197 (35.3)	24940.7 (35.4)	0.001
Multiple births	378 (3.2)	1822 (2.6)	0.036	378 (3.2)	2240.8 (3.2)	<0.001
Genitourinary tract infection	1270 (10.7)	6146 (8.7)	0.067	1270 (10.7)	7510.5 (10.7)	0.001
Sexually Transmitted Infection	188 (1.6)	913 (1.3)	0.024	188 (1.6)	1123.6 (1.6)	0.001
Treatment of chronic medical conditions	2175 (18.3)	12110 (17.2)	0.029	2175 (18.3)	12935.9 (18.4)	0.001

*Exposure propensity scores were measured as the predicted probability of receiving macrolides versus penicillins, conditional on the maternal and pregnancy related characteristics included in this table. 50 Strata were created based on the distribution of the propensity score of macrolides group. Weights for the penicillins group were calculated according to the distribution of the macrolides group among the strata and were used to estimate adjusted baseline characteristics. A meaningful between-group imbalance was assessed by an absolute standardised difference (St.diff, the difference in means in units of standard deviation) of more than 0.1. Numbers in adjusted penicillins group were non-integer, because they were weighted based on the distribution of propensity score of macrolides group.

Table S8. Subgroup analysis according to macrolides subtypes, on the association between adverse child outcomes and macrolides versus penicillins prescribed during pregnancy.

Adverse Outcomes	No. of events		Risk per 1,000 live births or Rate per 1,000 person-year		Adj. RR/HR (95% CI)	*P* value
	Macrolides	Penicillins	Macrolides	Penicillins		
**Erythromycin**						
**Any major malformation**						
1st trimester	53	398	27.39	17.65	1.50 (1.13-1.99)	0.005
2nd -3rd trimester	112	1268	18.51	17.27	1.07 (0.88-1.29)	0.507
**Nervous system malformation**						
1st trimester	6	27	3.10	1.20	2.47 (1.03-5.96)	0.044
2nd -3rd trimester	5	70	0.83	0.95	0.84 (0.34-2.08)	0.706
**Cardiovascular malformation**						
1st trimester	19	149	9.82	6.61	1.48 (0.92-2.37)	0.108
2nd -3rd trimester	41	477	6.77	6.50	1.02 (0.74-1.41)	0.889
**Gastrointestinal malformation**						
1st trimester	<5	20	-	0.80	0.55 (0.07-4.09)	0.56
2nd -3rd trimester	10	67	1.65	0.91	1.75 (0.90-3.39)	0.099
**Genital malformation**						
1st trimester	10	68	5.17	3.02	1.62 (0.84-3.14)	0.151
2nd -3rd trimester	26	227	4.30	3.09	1.45 (0.96-2.17)	0.075
**Urinary malformation**						
1st trimester	<5	41	-	1.82	0.54 (0.13-2.22)	0.392
2nd -3rd trimester	8	105	1.32	1.43	0.96 (0.47-1.97)	0.906
**Cerebral palsy**						
1st trimester	<5	55	-	0.35	0.21 (0.03-1.56)	0.128
2nd -3rd trimester	15	118	0.35	0.22	1.49 (0.87-2.57)	0.147
**Epilepsy**						
1st trimester	12	160	0.89	1.02	0.88 (0.49-1.58)	0.663
2nd -3rd trimester	35	525	0.81	0.99	0.84 (0.59-1.18)	0.312
**ADHD**						
1st trimester	12	123	0.88	0.78	1.12 (0.61-2.04)	0.714
2nd -3rd trimester	31	392	0.72	0.74	0.97 (0.67-1.40)	0.868
**ASD**						
1st trimester	19	181	1.40	1.15	1.15 (0.71-1.84)	0.575
2nd -3rd trimester	48	595	1.11	1.12	0.99 (0.74-1.33)	0.937
**Clarithromycin**						
**Any major malformation**						
1st trimester	6	398	36.81	17.65	1.83 (0.83-4.04)	0.133
2nd -3rd trimester	11	1268	33.23	17.27	2.07 (1.15-3.71)	0.015

*The macrolides group included 7987 (clarithromycin), 494 (clarithromycin) and 151 (azithromycin) children. In accordance with the confidentiality preserving policy of CPRD, we suppressed the information where the frequency cell contains <5 events (noted as "<5") and where necessary to avoid deduction. For clarithromycin, we only analysed any major malformation due to the limited number of events of other adverse child outcomes (there were six events of the four neurodevelopmental disorders in total in children prenatally prescribed clarithromycin). 151 azithromycin were prescribed during the whole pregnancy with <5 events of malformation, which precluded the analyses. ADHD: attention-deficit/hyperactivity disorder; ASD: autism spectrum disorder; CI: confidence interval; RR: risk ratio; HR: hazard ratio.

Table S9. Subgroup analysis according to duration of treatment (< 7 days or ≥ 7 days), on the association between adverse child outcomes and macrolides versus penicillins prescribed during pregnancy.

Adverse Outcomes	No. of events		Risk per 1,000 live births or Rate per 1,000 person-year		Adj. RR/HR (95% CI)	*P* value
	Macrolides	Penicillins	Macrolides	Penicillins		
**Any major malformation**						
<7 days, 1st trimester	17	144	37.28	16.58	2.11 (1.27-3.51)	0.004
≥7 days, 1st trimester	33	231	23.98	18.34	1.34 (0.94-1.92)	0.104
<7 days, 2nd -3rd trimester	25	466	15.30	16.46	0.88 (0.59-1.33)	0.553
≥7 days, 2nd -3rd trimester	84	724	20.70	17.81	1.18 (0.94-1.47)	0.154
**Nervous system malformation**						
<7 days, 1st trimester	<5	11	-	1.27	0 (0-Inf)	0.991
≥7 days, 1st trimester	<5	14	-	1.11	1.96 (0.56-6.82)	0.289
<7 days, 2nd -3rd trimester	<5	26	-	0.92	1.18 (0.28-4.94)	0.820
≥7 days, 2nd -3rd trimester	<5	39	-	0.96	0.54 (0.13-2.24)	0.398
**Cardiovascular malformation**						
<7 days, 1st trimester	7	59	15.35	6.79	2.39 (1.10-5.23)	0.028
≥7 days, 1st trimester	13	82	9.45	6.51	1.45 (0.81-2.60)	0.207
<7 days, 2nd -3rd trimester	9	167	5.51	5.9	0.87 (0.44-1.69)	0.675
≥7 days, 2nd -3rd trimester	30	271	7.39	6.67	1.10 (0.75-1.60)	0.624
**Gastrointestinal malformation**						
<7 days, 1st trimester	<5	9	-	1.04	0 (0-Inf)	0.990
≥7 days, 1st trimester	<5	10	-	0.79	0.86 (0.11-6.69)	0.888
<7 days, 2nd -3rd trimester	<5	25	-	0.88	2.36 (0.71-7.87)	0.162
≥7 days, 2nd -3rd trimester	8	37	1.97	0.91	2.02 (0.95-4.32)	0.069
**Genital malformation**						
<7 days, 1st trimester	<5	25	-	2.88	1.22 (0.29-5.07)	0.787
≥7 days, 1st trimester	6	40	4.36	3.18	0.86 (0.11-6.69)	0.888
<7 days, 2nd -3rd trimester	6	93	3.67	3.28	2.36 (0.71-7.87)	0.162
≥7 days, 2nd -3rd trimester	20	127	4.93	3.12	2.02 (0.95-4.32)	0.069
**Urinary malformation**						
<7 days, 1st trimester	<5	13	-	1.50	1.55 (0.20-	0.674
≥7 days, 1st trimester	<5	26	-	2.06	0.76 (0.18-3.20)	0.704
<7 days, 2nd -3rd trimester	<5	44	-	1.55	0.39 (0.05-2.79)	0.345
≥7 days, 2nd -3rd trimester	8	56	1.97	1.38	1.45 (0.69-3.05)	0.322
**Cerebral palsy**						
<7 days, 1st trimester	<5	20	-	0.28	0 (0-0)	<0.001
≥7 days, 1st trimester	<5	31	-	0.39	0.60 (0.14-2.53)	0.487
<7 days, 2nd -3rd trimester	<5	56	-	0.23	0.31 (0.04-2.25)	0.246
≥7 days, 2nd -3rd trimester	13	57	0.50	0.21	1.98 (1.05-3.73)	0.034
**Epilepsy**						
<7 days, 1st trimester	<5	71	-	1.01	0.71 (0.22-2.26)	0.559
≥7 days, 1st trimester	5	81	0.57	1.02	0.51 (0.21-1.28)	0.154
<7 days, 2nd -3rd trimester	12	232	0.82	0.98	0.91 (0.51-1.64)	0.762
≥7 days, 2nd -3rd trimester	22	262	0.85	0.99	0.87 (0.56-1.35)	0.543
**ADHD**						
<7 days, 1st trimester	5	55	1.28	0.78	1.53 (0.61-3.87)	0.367
≥7 days, 1st trimester	8	61	0.91	0.77	1.24 (0.58-2.64)	0.573
<7 days, 2nd -3rd trimester	14	191	0.96	0.80	1.25 (0.73-2.15)	0.418
≥7 days, 2nd -3rd trimester	12	188	0.46	0.71	0.63 (0.35-1.13)	0.122
**ASD**						

<7 days, 1st trimester	5	74	1.28	1.06	1.43 (0.62-3.30)	0.406
≥7 days, 1st trimester	11	96	1.25	1.21	0.74 (0.38-1.45)	0.385
<7 days, 2nd -3rd trimester	14	257	0.96	1.08	0.93 (0.54-1.59)	0.786
≥7 days, 2nd -3rd trimester	32	299	1.24	1.13	1.07 (0.74-1.54)	0.715

*97772 (93.5%) children in the study cohort were with non-missing duration of treatment. The macrolides group included 456 (<7 days, 1st trimester), 1376 (≥7 days, 1st trimester), 1634 (<7 days, 2nd -3rd trimester) and 4058 (≥7 days, 2nd -3rd trimester) children. The penicillins group included 8683 (<7 days, 1st trimester), 12592 (≥7 days, 1st trimester), 28314 (<7 days, 2nd -3rd trimester) and 40659 (≥7 days, 2nd -3rd trimester) children. In accordance with the confidentiality preserving policy of CPRD, we suppressed the information where the frequency cell contains <5 events (noted as "<5") and where necessary to avoid deduction. Within macrolides prescription during the 1st trimester, 95% prescriptions less than 7 days were of 5-6 days, and 93% prescriptions >= 7 days were of 7 days. Overall, 94.7% macrolides or penicillins prescriptions were of 5 to 7 days. ADHD: attention-deficit/hyperactivity disorder; ASD: autism spectrum disorder; CI: confidence interval; RR: risk ratio; HR: hazard ratio.

Table S10. Sensitivity analysis: comparison of the risks (or hazards) between siblings of children prenatally prescribed macrolides and siblings of children prenatally prescribed penicillins in the study cohort, according to timing of prescription.

Adverse outcomes	No. of events in siblings of children prescribed		Risk per 1,000 live births or Rate per 1,000 person-year in siblings of children prescribed		Adj. RR/HR in siblings (95% CI)	*P* value
	Macrolides	Penicillins	Macrolides	Penicillins		
**Any major malformation**						
1st trimester	25	210	21.22	18.06	1.18 (0.78-1.78)	0.429
2nd -3rd trimester	65	665	19.50	17.69	1.10 (0.85-1.41)	0.479
**Nervous system malformation**						
1st trimester	<5	9	-	1.20	0 (0-inf)	0.990
2nd -3rd trimester	6	40	1.80	1.06	1.73 (0.73-4.07)	0.213
**Cardiovascular malformation**						
1st trimester	7	81	5.94	6.96	0.87 (0.40-1.88)	0.727
2nd -3rd trimester	23	230	6.90	6.12	1.12 (0.73-1.72)	0.598
**Gastrointestinal malformation**						
1st trimester	<5	20	-	1.72	0.44 (0.06-3.26)	0.422
2nd -3rd trimester	6	33	1.80	0.88	1.88 (0.79-4.46)	0.152
**Genital malformation**						
1st trimester	6	42	5.09	3.61	1.44 (0.61-3.37)	0.407
2nd -3rd trimester	13	139	3.90	3.70	1.06 (0.60-1.87)	0.844
**Urinary malformation**						
1st trimester	<5	10	-	0.86	4.08 (1.27-13.07)	0.018
2nd -3rd trimester	5	51	1.50	1.36	1.10 (0.44-2.75)	0.843
**Cerebral palsy**						
1st trimester	<5	20	-	0.21	0.46 (0.06-3.43)	0.448
2nd -3rd trimester	6	66	0.22	0.21	0.99 (0.42-2.29)	0.973
**Epilepsy**						
1st trimester	<5	81	0.32	0.84	0.35 (0.11-1.11)	0.075
2nd -3rd trimester	23	276	0.84	0.87	0.96 (0.62-1.47)	0.841
**ADHD**						
1st trimester	7	76	0.74	0.79	0.91 (0.42-1.98)	0.807
2nd -3rd trimester	22	264	0.80	0.83	0.99 (0.63-1.56)	0.973
**ASD**						
1st trimester	13	93	1.38	0.96	1.36 (0.76-2.42)	0.297
2nd -3rd trimester	52	369	1.90	1.16	1.59 (1.16-2.17)	0.004

*1178 (macrolides, 1st trimester), 11631 (penicillins, 1st trimester), 3334 (macrolides, 2nd-3rd trimester), and 37592 (penicillins, 2nd-3rd trimester) children were included in the analyses. In accordance with the confidentiality preserving policy of CPRD, we suppressed the information where the frequency cell contains <5 events (noted as "<5") and where necessary to avoid deduction. Higher risks for genital malformation were observed for the both groups in the sibling cohort for unknown reason. ADHD: attention-deficit/hyperactivity disorder; ASD: autism spectrum disorder; CI: confidence interval; RR: risk ratio; HR: hazard ratio.

Table S11. Sensitivity analysis on the association between adverse child outcomes and macrolides versus penicillins prescribed during pregnancy: restricting to mothers whose antibiotics were prescribed to respiratory tract infections.

Adverse Outcomes	No. of events		Risk per 1,000 live births or Rate per 1,000 person-year		Adj. RR/HR (95% CI)	*P* value
	Macrolides	Penicillins	Macrolides	Penicillins		
**Any major malformation**						
1st trimester	30	159	35.42	18.71	1.81 (1.24-2.66)	0.002
2nd -3rd trimester	43	462	16.00	16.52	0.99 (0.73-1.35)	0.944
**Nervous system malformation**						
1st trimester	<5	7	-	0.82	1.46 (0.18-11.88)	0.723
2nd -3rd trimester	<5	25	-	0.89	0.88 (0.21-3.73)	0.862
**Cardiovascular malformation**						
1st trimester	11	61	12.99	7.18	1.79 (0.94-3.38)	0.075
2nd -3rd trimester	16	187	5.95	6.69	0.91 (0.55-1.52)	0.723
**Gastrointestinal malformation**						
1st trimester	<5	7	-	0.82	1.27 (0.16-10.14)	0.823
2nd -3rd trimester	5	25	1.86	0.89	2.19 (0.84-5.75)	0.110
**Genital malformation**						
1st trimester	9	26	10.63	3.06	3.30 (1.56-6.99)	0.002
2nd -3rd trimester	10	72	3.72	2.57	1.49 (0.77-2.89)	0.235
**Urinary malformation**						
1st trimester	<5	16	-	1.88	1.24 (0.29-5.37)	0.775
2nd -3rd trimester	5	51	1.12	1.50	0.75 (0.23-2.41)	0.626
**Cerebral palsy**						
1st trimester	<5	25	-	0.40	0.46 (0.06-3.38)	0.444
2nd -3rd trimester	7	41	0.38	0.20	1.82 (0.81-4.08)	0.146
**Epilepsy**						
1st trimester	<5	53	-	0.85	0.62 (0.19-1.98)	0.418
2nd -3rd trimester	15	215	0.81	1.05	0.77 (0.45-1.30)	0.332
**ADHD**						
1st trimester	<5	47	-	0.76	0.70 (0.22-2.24)	0.543
2nd -3rd trimester	15	151	0.81	0.73	1.17 (0.69-1.98)	0.565
**ASD**						
1st trimester	6	80	1.04	1.29	0.75 (0.33-1.71)	0.491
2nd -3rd trimester	14	195	0.76	0.95	0.78 (0.45-1.34)	0.368

*In accordance with the confidentiality preserving policy of CPRD, we suppressed the information where the frequency cell contains <5 events (noted as “<5”) and where necessary to avoid deduction. ADHD: attention-deficit/hyperactivity disorder; ASD: autism spectrum disorder; CI: confidence interval; RR: risk ratio; HR: hazard ratio.

Text S3. Probabilistic multiple bias analysis on outcome misclassification and live- birth bias

The Clinical Practice Research Datalink (CPRD) has been used increasingly widely in pharmacoepidemiology studies within academic, regulatory, and pharmaceutical organisations to inform treatment guidelines and clinical practice guidance.(13) However, outcome measurements derived from administrative databases such as CPRD are not perfect and misclassification bias may exist. As CPRD data were prospectively collected as part of routine healthcare, it is reasonable to assume that measurement errors of outcomes were non-differential between macrolides and penicillins groups. This non-differential outcome misclassification is likely to bias the relative risk (RR) estimates towards the null.(14)

Besides, we included only pregnancies that resulted in live-born children, thus some severe adverse outcomes (e.g. nervous system, cardiovascular and gastrointestinal malformations) that result in fetal deaths were missed. This depletion of affected fetuses may occur more often among women exposed to macrolides (versus penicillins), as shown in our systematic review (15). Therefore, risk ratio of these outcomes measured only in live births would be subject to selection (live-birth) bias with unknown direction.

We thus conducted probabilistic multiple bias analyses to quantify the bias due to outcome misclassification as well as jointly with live-birth bias to facilitate interpretation. Specifically, we estimated adjusted RR (95% CI) for each adverse child outcome for first-trimester macrolides (versus penicillins) prescribing using bias parameters stemming from both previous studies and educated guess.

Multiple bias analyses (which provided bias-adjusted RR estimates using standard 2x2 tables) were described in detail elsewhere (16). Briefly, frequencies in the tables were adjusted by a set of bias parameters, i.e. sensitivity and specificity for outcome misclassification, and probability of live birth for selection bias. These parameters were randomly sampled from given probability distributions (e.g. 5,000 iterations from triangular distributions in this study). In each iteration, we adjusted for misclassification bias and selection bias by sampling and adjusting the frequencies sequentially, incorporated with a random error to obtain the adjusted estimates with 95% limits. The analyses were performed using RStudio version 3.5.1 and R package “episensr”.(17) The bias parameters used and bias-adjusted results were presented in Text S3. Table-1 and Text S3. Table-2, respectively.

Results show that given the assumptions described above, adjustment for the outcome misclassification and live-birth bias resulted in elevated RRs for malformations. The RR increased from 1.62 to 1.78 for cardiovascular malformations, and slightly from 1.55 to 1.58 for any major malformation. RRs for the nervous system and genital malformations increased and became statistically significant with wide 95% limits. The adjustment for outcome misclassification did not alter our findings for neurodevelopmental disorders.

Text S3. Table-1. Summary of Prior Distributions of the Bias Parameters for the Probabilistic Multiple Bias Analyses.

Parameters	Evidence on bias parameters	Distributions of bias parameters
**Outcome misclassification**		
Sensitivity	**Major malformations**: The CPRD primary care database was considered a more complete source to investigate major malformation compared with national malformation registry, because primary care follow up records for registered patients. In contrast, malformation registry data is based on voluntary reports and active follow-up which is subject to attrition.(18-21) Based on our data, the prevalence of major malformation and major cardiovascular malformation were 17.0 and 6.3 per 1000 by the age of 3, respectively. These prevalence rates were slightly higher than those reported by the European Surveillance of Congenital Anomalies (EUROCAT) UK estimates (15.3 and 4.3 per 1000). The prevalence of major cardiovascular malformation in our data was also consistent with other reports using CPRD, of 5.1 to 8.3 per 1000 from ages 1 to age 6 in CPRD.(19) Considering there would be a small portion of malformations diagnosed after age 3 years,(21) we hence assume a not perfect but high sensitivity of malformation in our study, e.g. 0.95, with the range from 0.90 to 1.	Triangular (0.90, 0.95, 1)*
	**Cerebral palsy**: The prevalence is from 2 to 2.5 per 1000 for the whole population in the UK.(22) We observed a prevalence of 1.8 per 1000 live births till age 14 in this study, and thus assumed a sensitivity from 0.70 to 0.90, with a mode of 0.80.	Triangular (0.70, 0.80, 0.90)
	**Epilepsy:** The prevalence is 7 to 8 per 1000 for the whole population in the UK.(23) We observed a prevalence of 6.2 per 1000 live births till age 14 in this study, and thus assumed a sensitivity from 0.78 to 0.89, with a mode of 0.84.	Triangular (0.78, 0.84, 0.89)
	**ADHD:** The prevalence estimates vary widely across studies. While the prevalence in screening studies using the Development and Well-Being Assessment (DAWBA) was 36 per 1000 boys and 9 per 1000 girls, studies based on CPRD reported much lower prevalence rates of ADHD ranging from 4.4 to 8.7 per 1000 boys, and 0.5 to 1.2 per 1000 girls. (9, 24, 25) We observed a prevalence of 7.5 per 1,000 boys and 1.4 per 1,000 girls in this study, comparable to other CPRD studies. The lower prevalence captured in primary care databases is not surprising though, as ADHD is believed to be an underdiagnosed and undertreated condition, with only 43.7%-54.1% children with current ADHD receiving medications in the US and UK.(26, 27) We assumed a sensitivity from 0.50 to 0.90, with a mode of 0.70.	Triangular (0.50, 0.70, 0.90)
	**ASD:** The prevalence is about 10 per 1000 for the whole population in the UK.(28) We observed a prevalence of 7.7 per 1,000 live births till age 14, and thus assumed a sensitivity from 0.77 to 1, with a mode of 0.89.	Triangular (0.77, 0.89, 1)
Specificity	Specificity is not commonly measured for rarer outcomes in CPRD. However, a high specificity for all outcomes was expected in this study, due to both the low prevalence and the high positive predictive value (PPV). The high PPV of diagnosis in CPRD has been addressed by a number of studies. The PPV for major malformations, including cardiovascular malformations and hypospadias, has been reported to be 93% to 96% (31-33). The identification criteria we used for neurodevelopmental disorders have also been validated by previous researches in UK's primary care databases.(9, 10, 29) We thus assume a PPV of 95% for all outcomes in general population.<br>Based on the definition of specificity,	Triangular (0.997, 0.999, 1)

	$Specificity = 1 - \frac{False\ positive}{True\ negative} = 1 - \frac{N_{Observed\ positive} \times (1 - PPV)}{N_{all} \times (1 - prevalence)}$ $= 1 - \frac{N_{Observed\ positive}}{N_{all}} \times \frac{0.05}{> 0.95} = 1 - (< 0.05) \times (\frac{0.05}{> .95}) > 0.997$<br><br>We then assume a specificity for all outcomes from 0.997 to 1, with a mode of 0.999.	
**Live-birth bias for the association between first trimester macrolides prescribing and severe malformations (i.e. nervous system malformation, cardiovascular malformation and gastrointestinal malformation)**		
**Probability of live-birth (selection)**	**P (live-birth|(non-malformed, penicillin)):** 0.83. Around 17% pregnancies were terminated with non-clinical indication.(30) We thus assumed that the probability of live birth in penicillins group without malformation was with a mode of 0.83, and a range of 10%.	Triangular (0.78, 0.83, 0.88)
	**P (live-birth|(malformed, penicillin)):** 0.63, 0.73 and 0.78 for nervous system malformation, cardiovascular malformation and gastrointestinal malformation respectively. Based on estimated risk of termination, stillbirth, and first day neonatal death among cases with specific malformations, we assume 20%, 10% and 5% of cases with nervous system malformation, cardiovascular malformation and gastrointestinal malformation were dead before registration with the general practice.(31) Therefore, the probability of live birth is estimated to be 1-17%-(20%, 10% or 5%)=63%, 73% or 78% for cases with these three malformations, respectively. We estimated a range of 10%.	Nervous system malformation: Triangular (0.58, 0.63, 0.68)<br>Cardiovascular malformation: Triangular (0.68, 0.73, 0.78)<br>Gastrointestinal malformation: Triangular (0.73, 0.78, 0.83)
	**P (live-birth|(non-malformed, macrolides))** = P (live birth|(non-malformed, penicillin))-10%=0.73. Based on our previous system review, where the pooled odds ratio for miscarriage between macrolides and penicillins was 1.82, we assumed that first trimester macrolides exposure would decrease the probability of live birth by up to 10% (based on a probability of miscarriage of 12% in penicillin group*82%), compared to penicillins in fetuses with or without malformation.(15)	Triangular (0.68, 0.73, 0.78)
	**P (live-birth|(malformed, macrolides))** = P (live birth|(malformed, penicillin))-10%=0.53, 0.63 and 0.68 for nervous system malformation, cardiovascular malformation and gastrointestinal malformation respectively.	Nervous system malformation: Triangular (0.48, 0.53, 0.58)<br>Cardiovascular malformation: Triangular (0.58, 0.63, 0.68)<br>Gastrointestinal malformation: Triangular (0.63, 0.68, 0.73)

*Triangular (min, mode, max): Triangular distribution with minimum value, mode and maximum value. ADHD: attention-deficit/hyperactivity disorder; ASD: autism spectrum disorder.

Text S3. Table-2. Risk ratios adjusted by propensity score, and adjusted by bias due to outcome misclassification and conditioning on live-birth with random error for first trimester macrolides (versus penicillins) prescribing.

Child adverse outcomes	No. of events		Adjusted risk ratio (95% CI)[b]	+ Adjust bias due to outcome misclassification with random error (95% limits)	+ Adjust bias due to live-birth bias with random error (95% limits)
	Macrolides	Penicillins[a]			
Any major malformation	60	400.7	1.55 (1.21, 2.03)	1.58 (1.22, 2.08)	
Nervous system malformation	6	27.1	2.30 (0.95, 5.55)	5.17 (1.53, 31.24)	5.64 (1.62, 104.15)
Cardiovascular malformation	23	146.9	1.62 (1.05, 2.51)	1.74 (1.11, 2.74)	1.78 (1.12, 2.80)
Gastrointestinal malformation	<5[c]	-	1.00 (0.23, 4.28)	1.04 (0.24, 4.31)	1.00 (0.23, 4.14)
Genital malformation	11	68.1	1.68 (0.89, 3.16)	2.04 (1.03, 3.94)	
Urinary malformation	<5[c]	-	0.65 (0.20, 2.08)	0.49 (0.14, 1.62)	
Cerebral palsy	<5[c]	-	0.39 (0.10, 1.61)	0.27 (0.06, 1.15)	
Epilepsy	12	160.4	0.78 (0.43, 1.39)	0.74 (0.41, 1.30)	
ADHD	14	122.1	1.19 (0.69, 2.06)	1.24 (0.71, 2.16)	
ASD	19	198.9	0.99 (0.62, 1.58)	0.99 (0.60, 1.56)	

a: The numbers of event in penicillins group were weighted based on the distribution of propensity score of macrolides group, which were used to calculate the adjusted risk/hazard ratio in the main analyses. b: Because the risk ratios for cerebral palsy, epilepsy, ADHD and ASD were comparable with the reported hazard ratios, we measured their risk ratios for simplicity. c: In accordance with the confidentiality preserving policy of CPRD, we suppressed the information where the frequency cell contains <5 events (noted as "<5") and where necessary to avoid deduction. CI: confidence interval; RR: risk ratio; HR: hazard ratio. ADHD: attention-deficit/hyperactivity disorder; ASD: autism spectrum disorder

Table S12. ***Post-hoc*** **analyses on the association between common specific malformation and macrolides versus penicillins prescribed during pregnancy.**

Adverse Outcomes	No. of events		Risk per 1,000 live births or Rate per 1,000 person-year		Adj. RR/HR (95% CI)	P value
	Macrolides	Penicillins	Macrolides	Penicillins		
**Ventricular septal defect**						
1st trimester	13	85	5.99	3.77	1.66 (0.93-2.98)	0.088
2nd -3rd trimester	25	252	3.87	3.43	1.11 (0.73-1.67)	0.626
**Hypospadias***						
1st trimester	10	61	8.86	5.26	1.45 (0.75-2.81)	0.268
2nd -3rd trimester	26	206	8.16	5.47	1.56 (1.04-2.35)	0.032
**Atrial septal defect**						
1st trimester	5	26	2.3	1.15	2.01 (0.77-5.22)	0.154
2nd -3rd trimester	5	89	0.77	1.21	0.59 (0.24-1.44)	0.244
**Patent ductus arteriosus**						
1st trimester	<5	40	-	1.77	0.84 (0.26-2.74)	0.778
2nd -3rd trimester	12	127	1.86	1.73	1.02 (0.57-1.84)	0.946
**Cleft palate/lip**						
1st trimester	<5	29	-	1.29	0.75 (0.18-3.14)	0.692
2nd -3rd trimester	11	94	1.7	1.28	1.29 (0.69-2.40)	0.425
**Craniosynostosis**						
1st trimester	<5	5	-	0.22	4.16 (0.81-21.45)	0.088
2nd -3rd trimester	5	14	0.77	0.19	3.87 (1.40-10.67)	0.009

*Calculated in male babies. In accordance with the confidentiality preserving policy of CPRD, we only analyses outcomes where there were at least 5 cases in 1st trimester or 2nd to 3rd trimester, macrolides group. We suppressed the information where the frequency cell contains <5 events (noted as "<5") and where necessary to avoid deduction. CI: confidence interval; RR: risk ratio; HR: hazard ratio.

Table S13. Number of prescriptions matched or not matched with any indication (infection) and number of any major malformation by each indication.

No. of prescriptions matched or not matched with indication (infection)		Macrolides				Penicillins	No. of any major malformation	
		Total	Erythromycin	Clarithromycin	Azithromycin		Macrolides	Penicillins
Antibiotics matched with any indication		4726 (55%)	4366 (55%)	287 (58%)	73 (48%)	52293 (54%)	94 (1.99%)	915 (1.75%)
**Indication**	Respiratory tract infection	3534 (75%)	3298 (76%)	224 (78%)	12 (16%)	36462 (70%)	73 (2.07%)	621 (1.70%)
	Skin infection	377 (8%)	363 (8%)	-	<5	3019 (6%)	9 (2.39%)	65 (2.15%)
	Head & Neck infection	306 (6%)	274 (6%)	-	<5	2979 (6%)	6 (1.96%)	51 (1.71%)
	Genitourinary infection	197 (4%)	191 (4%)	<5	<5	9515 (18%)	<5	173 (1.82%)
	Sexual transmitted infection	163 (3%)	107 (2%)	<5	-	63 (0%)	<5	<5
	Gastrointestinal infection	135 (3%)	121 (3%)	-	<5	171 (0%)	<5	5 (2.92%)
	Other infections	14 (0%)	12 (0%)	<5	<5	84 (0%)	<5	<5
Antibiotics unmatched with any indication		3906 (45%)	3621 (45%)	207 (42%)	78 (52%)	43680 (46%)	92 (2.36%)	751 (1.72%)
**Total**		**8632**	**7987**	**494**	**151**	**95973**	**186**	**1666**

*An indication was defined as an infection episode recorded within 6 days before a macrolide or penicillin prescription. In accordance with the confidentiality preserving policy of CPRD, we suppressed the information where the frequency cell contains <5 events (noted as “<5”) and where necessary to avoid deduction.

Table S14. Previously published studies on the association between maternal exposure of macrolides and major congenital malformations or neurodevelopmental disorders.

Studies	Study type	Exposure	Reference group	Outcome	No. of cases/Total in exposure group	RR/OR (95% Confidence interval)	Comments
Einarson, 1990	Prospective cohort	Clarithromycin, 4-14 weeks	Non-teratogenic antibiotics	Major CM	3/157	1.60 (0.26-9.69)	
Czeizel,1999	Paired case-control, Hungarian	Erythromycin, 2-3 month and whole pregnancy	Non-exposure to erythromycin	Isolated CMs	23 cases	1.50 (0.80-2.60) for Cardiovascular CA	
Kallen, 2005	Swedish Medical Birth Register	Erythromycin, 1st trimester	General population and indirectly penicillin V	Cardiovascular CM	31/1844	1.84 (1.29-2.62)	Penicillins Versus general population: 0.99 (0.80-1.23)
Sakar, 2006	Prospective cohort, Canada	Azithromycin, whole pregnancy	Antibiotics, Non-teratogens	Major CM	3/123	Not reported	Under power
Kenyon, 2008	Randomised Clinical Trial	Erythromycin + co-amoxiclav or erythromycin only, 3rd trimester	co-amoxiclav or placebo	Cerebral palsy	18/783 in pPROM, 35/795 in SPL	0.91 (0.48-1.71) in pPROM. 2.28 (1.24-4.21) in SPL	
Kenyon, 2008	Randomised Clinical Trial	Erythromycin + co-amoxiclav or erythromycin only, 3rd trimester	co-amoxiclav or placebo	Epilepsy	18/783 in pPROM, 35/795 in SPL	0.89 (0.59-1.32) in pPROM, 1.18 (0.84-1.66) in SPL	
Cooper, 2009	Tennessee Medicaid	Erythromycin, azithromycin, first 4 lunar months	No antibiotics	Major and system CM	23 major CM/903 in erythromycin group; 23 major CM/559 in azithromyci(32)n group	0.86 (0.55-1.34) for erythromycin, major CM; 1.37 (0.85-2.22) for azithromycin, major CM	
Crider, 2009	case-control, Hungarian	Erythromycin, whole pregnancy	No erythromycin	Selected Birth Defects	>300 CM case in total	Anencephaly 2.4 (1.1-5.3) and transverse limb deficiency 2.1(1.0-4.2)	Associations with other outcomes were not significant. Any heart defect 1.0 (0.7-1.3).
Bar-Oz, 2012	Prospective cohort, Czech	Macrolides (Clarithromycin, azithromycin and roxithromycin), 1st trimester	Non-teratogenic exposures	Major and cardiovascular CM	15/441 (Major CM); 7/441 (cardiovascular CM)	1.42 (0.70, 2.88) for macrolides and major CM;<br>1.91 (0.63, 5.62) for macrolides and cardiovascular CM	
Romoren, 2012	Medical Birth Registry of Norway	Macrolides, 1ST trimester	Penicillin V	Major and cardiovascular CM	69/2549 (Major CM); 25/2549 (cardiovascular CM)	0.96 (0.76,1.22) for major CM;<br>0.96 (0.65,1.43) for cardiovascular CM	Gestational week 5-8: 1.36 (0.75, 2.47) for cardiovascular CM.
Andersen, 2013	Danish Fertility Database	Clarithromycin, 1ST trimester	No clarithromycin	Major CM	9/253	1.03 (0.53–2.00)	
Bahat, 2013	Retrospective cohort, Israel	Macrolides, 1st and 3rd trimester	No macrolides	Major and cardiovascular CM	Number of cases unreported, 1033 macrolides in total.	1.07 (0.84–1.38) for major CM;<br>0.95 (0.65–1.40) for cardiovascular CM	

Lin, 2013	Case-control, Slone Epidemiology Center Birth Defects Study	Macrolides and Erythromycin, 1-3 trimester	No erythromycin	Cardiovascular malformation	140 Cardiovascular CM cases in total	0.9 (0.6-1.3) for cardiovascular CM exposed to macrolides during 1st trimester	
Berard, 2015	Prospective cohort, Quebec Pregnancy Cohort	Erythromycin, azithromycin, and clarithromycin, 1st trimester	Unexposed	Major and cardiovascular CM	66/734 erythromycin, 120/914 azithromycin, and 79/686 clarithromycin.	0.96 (0.74–1.24) erythromycin, 1.19 (0.98–1.44) azithromycin and 1.12 (0.99–1.42) clarithromycin	
Meeraus, 2015	Retrospective cohort, UK	Macrolides, whole pregnancy	Penicillins	Cerebral palsy or epilepsy	28/2749	1.78 (1.18-2.69)	
Muanda, 2017	Prospective cohort, Quebec Pregnancy Cohort	Macrolides, 1st trimester	Penicillins	Major and system CM	265/2332 major CM, 35/2332 gastrointestinal CM, 18/2332 genital tract CM	1.13 (0.98–1.31) for major CM, 1.48 (0.99–2.20) for gastrointestinal CM, and 0.93 (0.55–1.56) for genital tract CM	Associations with other outcomes were not significant. High prevalence of major CM, though the author argued this is non-differential between exposure groups.

*CM: congenital malformation; pPROM: preterm rupture of the membranes; SPL: spontaneous preterm labour

Reference

1. Olsen IE, Groveman SA, Lawson ML, Clark RH, Zemel BS. New Intrauterine Growth Curves Based on United States Data. *Pediatrics*. 2010;125(2):e214-e24.
2. Matcho A, Ryan P, Fife D, Gifkins D, Knoll C, Friedman A. Inferring pregnancy episodes and outcomes within a network of observational databases. *PLoS One*. 2018;13(2):e0192033.
3. Office for National Statistics. Gestation-specific Infant Mortality in England and Wales, 2007-2008. In: Office for National Statistics, editor. 2014.
4. Minassian C, Williams R, Meeraus WH, Smeeth L, Campbell OMR, Thomas SL. Methods to generate and validate a Pregnancy Register in the UK Clinical Practice Research Datalink primary care database. *Pharmacoepidemiol Drug Saf*. 2019;28(7):923-33.
5. European Surveillance of Congenital Anomalies. The EUROCAT Guide: European Surveillance of Congenital Anomalies; [May 26th, 2019]. Available from: http://www.eurocat-network.eu/.
6. Sewell MD, Rosendahl K, Eastwood DM. Developmental dysplasia of the hip. *BMJ*. 2009;339:b4454.
7. European Surveillance of Congenital Anomalies. EUROCAT Prevalence Data Tables: European Surveillance of Congenital Anomalies; [May 26th, 2019]. Available from: http://www.eurocat-network.eu/.
8. Fan H, Li L, Gilbert R, O'Callaghan F, Wijlaars L. A machine learning approach to identify cases of cerebral palsy using the UK primary care database. *The Lancet*. 2018;392:S33.
9. Holden S, Jenkins-Jones S, Poole C, Morgan C, Coghill D, Currie C. The prevalence and incidence, resource use and financial costs of treating people with attention deficit/hyperactivity disorder (ADHD) in the United Kingdom (1998 to 2010). *Child Adolesc Psychiatry Ment Health*. 2013;7(1):34.
10. Hagberg KW, Jick SS. Validation of autism spectrum disorder diagnoses recorded in the Clinical Practice Research Datalink, 1990-2014. *Clin Epidemiol*. 2017;9:475-82.
11. Meeraus WH. Adverse Paediatric Outcomes of Antibiotic Treatment in Pregnancy: University College London; 2015.
12. Bell S, Daskalopoulou M, Rapsomaniki E, George J, Britton A, Bobak M, et al. Association between clinically recorded alcohol consumption and initial presentation of 12 cardiovascular diseases: population based cohort study using linked health records. *BMJ*. 2017;356:j909.
13. Ghosh RE, Crellin E, Beatty S, Donegan K, Myles P, Williams R. How Clinical Practice Research Datalink data are used to support pharmacovigilance. *Ther Adv Drug Saf*. 2019;10:2042098619854010.
14. Greenland S. Basic methods for sensitivity analysis of biases. *Int J Epidemiol*. 1996;25(6):1107-16.
15. Fan H, Gilbert R, Li L, Wijlaars L. Associations between use of macrolide antibiotics during pregnancy and adverse child outcomes: a systematic review and meta-analysis *Lancet*. 2018.
16. Lash T, Fox M, Fink A. Applying Quantitative Bias Analysis to Epidemiologic Data. 2009.
17. Haine D. episensr: Basic Sensitivity Analysis of Epidemiological Results: Denis Haine; 2019 [Available from: https://CRAN.R-project.org/package=episensr.
18. Wurst KE, Ephross SA, Loehr J, Clark DW, Guess HA. The utility of the general practice research database to examine selected congenital heart defects: a validation study. *Pharmacoepidemiol Drug Saf*. 2007;16(8):867-77.
19. Wurst KE, Ephross SA, Loehr J, Clark DW, Guess HA. Evaluation of the General Practice Research Database congenital heart defects prevalence: comparison to United Kingdom national systems. *Birth defects research Part A, Clinical and molecular teratology*. 2007;79(4):309-16.
20. Hammad TA, Margulis AV, Ding Y, Strazzeri MM, Epperly H. Determining the predictive value of Read codes to identify congenital cardiac malformations in the UK Clinical Practice Research Datalink. *Pharmacoepidemiol Drug Saf*. 2013;22(11):1233-8.

21. Sokal R, Fleming KM, Tata LJ. Potential of general practice data for congenital anomaly research: Comparison with registry data in the United Kingdom. *Birth defects research Part A, Clinical and molecular teratology*. 2013;97(8):546-53.
22. National Institute for Health and Care Excellence. Cerebral palsy in under 25s: assessment and management. In: National Institute for Health and Care Excellence, editor. London2017.
23. National Institute for Health and Care Excellence. The Epilepsies: The diagnosis and management of the epilepsies in adults and children in primary and secondary care. In: National Institute for Health and Care Excellence, editor. London2012.
24. Ford T, Goodman R, Meltzer H. The British Child and Adolescent Mental Health Survey 1999: The Prevalence of DSM-IV Disorders. *Journal of the American Academy of Child & Adolescent Psychiatry*. 2003;42(10):1203-11.
25. Hire AJ, Ashcroft DM, Springate DA, Steinke DT. ADHD in the United Kingdom: Regional and Socioeconomic Variations in Incidence Rates Amongst Children and Adolescents (2004-2013). *J Atten Disord*. 2018;22(2):134-42.
26. Danielson ML, Visser SN, Gleason MM, Peacock G, Claussen AH, Blumberg SJ. A National Profile of Attention-Deficit Hyperactivity Disorder Diagnosis and Treatment Among US Children Aged 2 to 5 Years. *Journal of developmental and behavioral pediatrics : JDBP*. 2017;38(7):455-64.
27. Bushe C, Wilson B, Televantou F, Belger M, Watson L. Understanding the treatment of attention deficit hyperactivity disorder in newly diagnosed adult patients in general practice: a UK database study. *Pragmat Obs Res*. 2015;6:1-12.
28. National Institute for Health and Clinical Excellence. Autism: recognition, referral and diagnosis of children and young people on the autism spectrum. In: National Institute for Health and Clinical Excellence, editor. London2011.
29. Meeraus WH, Petersen I, Chin RF, Knott F, Gilbert R. Childhood epilepsy recorded in primary care in the UK. *Archives of disease in childhood*. 2013;98(3):195-202.
30. Mortensen LH, Catalano RA, Bruckner TA. Spontaneous Pregnancy Loss in Denmark Following Economic Downturns. *American Journal of Epidemiology*. 2016;183(8):701-8.
31. Heinke D. An Evaluation of Competing Risks in Studies of Perinatal Mortality and Birth Defects. Boston: Harvard University; 2018.
32. Bar-Oz B, Weber-Schoendorfer C, Berlin M, Clementi M, Di Gianantonio E, De Vries L, et al. The outcomes of pregnancy in women exposed to the new macrolides in the first trimester: A prospective, multicentre, observational study. *Drug safety*. 2012;35(7):589-98.
